# Evidence-Based Challenges to the Continued Recommendation and Use of Peroxidatively-Susceptible Polyunsaturated Fatty Acid-Rich Culinary Oils for High-Temperature Frying Practises: Experimental Revelations Focused on Toxic Aldehydic Lipid Oxidation Products

**DOI:** 10.3389/fnut.2021.711640

**Published:** 2022-01-05

**Authors:** Martin Grootveld

**Affiliations:** Leicester School of Pharmacy, De Montfort University, Leicester, United Kingdom

**Keywords:** lipid oxidation products, thermal instability of PUFAs, aldehydes, aldehyde toxicity, culinary frying oils, fried foods, French fries, non-communicable chronic diseases

## Abstract

In this manuscript, a series of research reports focused on dietary lipid oxidation products (LOPs), their toxicities and adverse health effects are critically reviewed in order to present a challenge to the mindset supporting, or strongly supporting, the notion that polyunsaturated fatty acid-laden frying oils are “safe” to use for high-temperature frying practises. The generation, physiological fates, and toxicities of less commonly known or documented LOPs, such as epoxy-fatty acids, are also considered. Primarily, an introduction to the sequential autocatalytic peroxidative degradation of unsaturated fatty acids (UFAs) occurring during frying episodes is described, as are the potential adverse health effects posed by the dietary consumption of aldehydic and other LOP toxins formed. In continuance, statistics on the dietary consumption of fried foods by humans are reviewed, with a special consideration of French fries. Subsequently, estimates of human dietary aldehyde intake are critically explored, which unfortunately are limited to acrolein and other lower homologues such as acetaldehyde and formaldehyde. However, a full update on estimates of quantities derived from fried food sources is provided here. Further items reviewed include the biochemical reactivities, metabolism and volatilities of aldehydic LOPs (the latter of which is of critical importance regarding the adverse health effects mediated by the inhalation of cooking/frying oil fumes); their toxicological actions, including sections focussed on governmental health authority tolerable daily intakes, delivery methods and routes employed for assessing such effects in animal model systems, along with problems encountered with the Cramer classification of such toxins. The mutagenicities, genotoxicities, and carcinogenic potential of aldehydes are then reviewed in some detail, and following this the physiological concentrations of aldehydes and their likely dietary sources are considered. Finally, conclusions from this study are drawn, with special reference to requirements for (1) the establishment of tolerable daily intake (TDI) values for a much wider range of aldehydic LOPs, and (2) the performance of future nutritional and epidemiological trials to explore associations between their dietary intake and the incidence and severity of non-communicable chronic diseases (NCDs).

## Introduction

The Western diet contains large quantities of lipids which have been exposed to high temperature frying, cooking or processing episodes, and according to today's standards, consumers have expressed a high level of public health concern relating to the inherence, distribution and dietary consumption of adversely generated toxins and contaminants in foods (1). These concerns are perpetuated by reports, media, blogs, or otherwise, which associate the risks of developing or perpetuating non-communicable chronic diseases (NCDs), for example cancer (2) and cardiovascular diseases (3), with an excessive consumption of fried foods.

A summative assessment of the range of molecularly-destructive reactions occurring throughout the course of shallow- or deep-frying episodes, and some other forms of cooking episodes which involve the exposure of culinary oils to high temperatures (for example, wok cooking regimens) are outlined below. In general, the more important of these are classified in this list, but further minor thermally-induced processes are also of importance, most notably if any products arising therefrom have the potential to exert toxicological effects in humans.

- Peroxidation of unsaturated fatty acids (UFAs)
- Epoxy-fatty acids (FAs)
- Co-oxidation of lipids with food proteins
- Thermal degradation products generated during very high-temperature frying practises
- FA oligomerization and polymerization
- Triacylglycerol (TAG) hydrolysis products/polar compounds.

However, the primary focus of this toxicological assessment study is aldehydic LOPs as secondary, and sometimes tertiary LOPs arising from the peroxidation of UFAs, most especially those present in fried foods. Nevertheless, short to moderate length outlines of the generation, levels, biomolecular chemistry and potential toxicological effects exerted by the other major frying oil degradation products noted above are made in this section.

### Peroxidative Degradation of UFAs to Form Toxic Secondary LOPs Such as Aldehydes

The oxidative deterioration of unsaturated fatty acids (UFAs) present in cooking oils during standard high-temperature frying practises (4, 5) is probably the most important free radical-mediated and molecularly-degradative reaction process taking place during the course of such activities, and serves as the principal source of toxic lipid oxidation products (LOPs), which, together with the oil in which they are generated, penetrate foods fried therein, most commonly potato chips, or French fries, eggs, fish and meat patties, etc. It is also one of the most complex reaction schemes, and features a range of differential lipid precursors, primary, secondary and even tertiary LOPs, and many intermediates, side-reactions and by-products. Primary LOPs include highly toxic conjugated hydroperoxydienes (CHPDs) from polyunsaturated fatty acid (PUFA) peroxidation (and also hydroperoxymonoenes (HPMs) from monounsaturated fatty acid (MUFA) peroxidation), but at high frying temperatures (*ca*. 180°C), these are fragmented to a series of lower molecular mass secondary LOP compounds, most notably aldehydes, both saturated and unsaturated, which in turn may exert a wide range of deleterious toxicological actions if dietarily consumed or inhaled by humans (6–8). Further LOP compounds generated include hydrocarbons, ketones and carboxylic acids (6). Some aldehydes may be generated from the thermally-induced oxidation or degradation of other aldehydic LOPs formed secondarily, and for the purpose of the current paper, these are classified as tertiary aldehydic LOPs. PUFA peroxidation, along with the deterioration of their primary CHPDs to corresponding fragmentation products, are much more favourable and rapid processes than those observed for MUFAs, and therefore in a hypothetical frying oil containing exactly equimolar amounts of PUFAs and MUFAs, the peroxidation products arising from thermal stressing according to standard frying practises would mainly, but not exclusively, be derived from PUFA sources. In contrast, saturated fatty acids (SFAs) are almost completely resistant to this process (7–9). Furthermore, PUFAs generate a much wider pattern of different saturated and unsaturated aldehydic LOP classes (the latter group including alka-2,4-dienals (both *trans,trans-* and *cis,trans-* isomers) 4-hydroperoxy-/4-hydroxy-*trans*-2-alkenals and 4,5-epoxy-*trans*-2-alkenals), whereas those arising from MUFA peroxidation are largely limited to only *n*-alkanals and *trans*-2-alkenals (4–8).

Our previous NMR-based investigations have clearly demonstrated the peroxidative deterioration of culinary oil UFAs when exposed to standard or laboratory-simulated high-temperature frying practises (5, 6), information which was originally made available in 1994 (10). Since that time, these results have been ratified by many other researchers globally, e.g. (11), reaching out to active laboratories based in the North and South Americas, Australasia, Asia and Africa, as well as Europe.

Following their oral intake, chemically-reactive α,β-unsaturated aldehydes generated in such thermally-stressed PUFA-rich cooking oils have been demonstrated to be absorbed from the gut into the systemic circulation *in vivo* (12). To date, there is now an almost overwhelming series of scientific literature reports available on the toxic and adverse health effects of LOPs. Moreover, a similar volume of reports which associate increased risks of NCDs such as selected cancers and cardiovascular diseases with an excessive dietary consumption of fried foods, are also readily accessible. Indeed, aldehydic LOPs bolster a very wide range of deleterious, concentration-dependent health effects, and an inexhaustive summary of these includes the induction and proliferation of atherosclerosis and its cardiovascular disease sequelae (12–14); mutagenic and carcinogenic effects (15, 16); neurotoxic properties, particularly for formaldehyde, acetaldehyde, and 4-hydroxy-*trans*-2-nonenal (HNE) and -hexenal (HHE) (17); the exertion of astonishing pro-inflammatory effects at very low concentrations (18, 19); very concerning teratogenic actions (20); and gastropathic properties (peptic ulcers) subsequent to their dietary intake (21). These reports clearly provide powerful evidence for potential public health threats induced and perpetuated by LOPs present as food-borne toxins.

### Epoxy-Fatty Acids

A series of highly toxic epoxy-fatty acid (epoxy-FA) derivatives can arise as either primary or secondary lipid peroxidation pathways (22, 23). Epoxy-fatty acids are commonly generated in oils and fats when exposed to high-temperature frying episodes. Furthermore, they are also found in unheated foods such as pumpkin seed oil and chocolate. Frying practises involving UFA-containing culinary oils generate very high levels of monoepoxy-FAs, i.e., those over and above 7 g/kg (24, 25), which for monoepoxy-linoleic acid is equivalent to ~22 mmol./kg.

Velasco et al. (26) monitored the production and evolution of monoepoxy-FAs derived from oleoyl- and linoleoylglycerols present in olive and sunflower oils throughout thermo-oxidation periods of 5, 10, and 15 h. at 180°C. For this purpose, six monoepoxy-FAs from these derivatives were analysed by GC following their derivatization to FA methyl esters. Following exposure to these thermal-stressing episodes, monoepoxy-FA levels determined ranged from 4.3 to 14.2 g/kg in olive oil, and from 5.1 to 9.4 g/kg in sunflower oil, these products representing the major epoxy-FA products arising from the thermo-oxidation of these unsaturated acylglycerols at this temperature. However, overall there were higher contents of these products in olive rather than sunflower oil. The monoepoxy-FA contents of used oils collected from 10 restaurants and fried-food outlets in Spain were found to contain concentrations varying from 3.4 to 14.4 g/kg of oil. Hence, at similar levels of thermal degradation, these epoxy-FA toxins were found to be more prevalent in MUFA- rather than in PUFA-rich culinary oils.

The generation of monoepoxy-FAs in frying oils when exposed to high-temperature frying practises, particularly *trans*-9,10- and *cis*-9,10-epoxystearates from oleoylglycerols, and *trans*-12,13, *trans*-9,10-, *cis*-12,13- and *cis*- 9,10-epoxyoctadecenoates from linoleoylglycerol peroxidations, have been determined in used frying oils and fats at mean total levels as high as 3.7 g/kg (i.e., as much as 0.37 weight %), and also in the lipid phase of chocolate (at *ca*. 2 g/kg). Total levels of these toxins have also been determined in olive, sunflower, groundnut, sweet almond, and pumpkin seed oils at mean levels of 0.2, 1.0, 1.4, 1.7, and 3.4 g/kg, respectively (26), and hence, with the exception of olive oil, these contents do not appear to be dependent on their [PUFA]:[MUFA] molar concentration ratios. The generation of these oxidation products during exposure to thermal-stressing episodes at 175°C was notable for sunflower, rapeseed, soybean, and linseed oils, and these levels were correlated with increases observed in these oils' polar compound and polymerized triacylglycerol contents, which serve as further important quality measures for frying oils. During heating for a 16 h. duration, the total epoxy-FA concentrations of monoepoxy-FAs was elevated to ~4, 8, 18, and 30 g/kg for rapeseed, linseed, sunflower and olive oils, respectively. Moreover, the effects exerted by heating temperature was explored for refined soybean oil at 160, 170, 180, and 200°C, and it was found that on completion of the heating period (22 h), the content of total monoepoxy-FAs was 3-fold greater at the highest temperature explored (200°C) than it was at 160°C. Astonishingly, this 200°C experimental concentration was as high as 21.6 g/kg (nearly 70 mmol./kg).

Interestingly, subjection of two sets of five different refined and virgin rapeseed oils to heating episodes showed a significantly lowered generation of total monoepoxy-FAs in refined oils than those found in virgin products (median levels of 4.6 and 7.7 g/kg, respectively), although it was not clear why exactly. The authors of this report have proposed that a limit of 7 g/kg of total monoepoxy-FAs should be employed as a level for the usability screening of used frying oils and fats, most notably because all oils evaluated with these toxins at or above this limit had to be rejected for future use in view of pre-set, regulatory limits for polar compounds and triacylglycerol polymers being exceeded. However, this does seem a little excessive, especially on consideration of the adverse health effects potentially exerted by these toxins.

In a quantitative exposure evaluation using both deterministic and probabilistic strategies for combining concentration and consumption datasets from the Belgian National Food Consumption Survey (2004), Mubiri et al. (27) preliminarily explored the significance of any potential risks arising from the intake of epoxy-FAs via application of the threshold of toxicological concern (TTC) concept. Out of a total of 17 different food sources studied, mayonnaise, butter–margarine and ready-to-eat meals were found to represent the highest contributors to epoxy-FA intake. From the TTC strategy employed, risks to human health were identified regarding the dietary consumption of French fries, vegetable oils, cheese, snack foods, dry nuts, potato chips (“crisps”), cured raw minced meat and biscuits, together with both fresh and frozen salmon and bacon.

In 2006, Fankhauser-Noti et al. (28) explored the migration of epoxidized soybean oil (ESBO) into foods. Indeed, ESBO was found to exert toxicological effects when administered to rats, although for this study, the major toxic agent involved was not identified. This toxicological assessment was considered to be of critical importance, since the contact of edible oils and oily foods has been shown to extract epoxy-FAs from polyvinyl chloride (PVC) gaskets within the lids employed for closing jars used for the storage and sale of such foods. The amount liberated from these sources regularly far exceeds the European legal limit [overall migration limit and specific migration limit derived from the tolerable daily intake (TDI)]. Indeed, the mean amount of epoxidized soybean oil was as high as 166 mg/kg, with levels reaching a maximum of 580 mg/kg. Foods in the authors' EU countries which contained the highest epoxy-FA contents are thermally-stressed and degraded frying oils present in fried foods, and bakery and roasted meat products. However, the contribution of monoepoxy-oleate and -oleoylglycerols from epoxidized soybean oil to this diet was found to be negligible. Hence, the authors of this work suggested that if this agent was the only toxic component of these epoxidized oils, the toxicological assessment arising therefrom would, at least primarily, represent a simple “warning” regarding oxidised oils and fats. These authors also stress that since the contribution of diepoxy-linoleic acid from epoxidized soybean oil may resemble that arising from exposure to peroxidized dietary oils and fats anyway, and that the human intake of triepoxy-linolenic acid from this epoxidized oil exceeds that usually dietarily-derived by *ca*. two orders of magnitude, the consumption of an epoxidized edible oil which is linoleoylglcerol-free would be toxicologically insignificant in this diet. However, it appears that both monoepoxy-linoleic, and monoepoxy- and diepoxy-linolenic acid species, were not considered during the authors' summations, and potentially higher levels of these species (perhaps in decreasing order for linolenoylglycerols), would be expected to be generated in thermally-stressed culinary oils than the fully epoxidized products evaluated by these researchers.

The enzyme-soluble epoxide hydrolase (sEH) has been suggested to modify the mode of action and enhance the toxicological potential of epoxy-FAs (29). In order to establish the structural features required for the sEH-inducible epoxy-FA toxicity, a total of 75 compounds were screened for their cytotoxic actions, both with and without sEH, in the assay medium. Overall, data acquired supported the postulate that the methyl eaters of long-chain epoxy-FAs act as pro-toxins which are potentially metabolised to more toxic diol compounds. Moreover, the endogenous agents leukotoxin methyl ester, 9,10(*Z*)-epoxyoctadec-12(*Z*)-enoic acid methyl ester, and isoleukotoxin methyl ester, and 12,13(*Z*)-epoxyoctadec-9(*Z*)-enoic acid methyl ester were found to be molecularly-optimised to exert such toxic actions.

Finally, in 2002 Wilson et al. (30) investigated the *in vivo* absorption of epoxy-FAs in healthy women, and found that the monoepoxy- derivatives were absorbed more so than the diepoxy- ones (17 vs. 8%, *p* = 0.02); this difference was considered to be of much importance regarding the relative toxicological effects of these oxidation products, a realm of which is present in the food chain.

### Co-oxidation of Lipids With Food Proteins and DNA

For the investigation of agents which cause structural damage to proteins and DNA, it is, of course necessary to consider oxidants or reactants in general other than CHPDs and their fragmentation products when conducting an analysis of foods, food frying media, and physiological tissues, so that co-oxidation products are also included. Indeed, peroxidation products detectable at high levels in frying oils and foods fried therein comprise a wealth of such molecularly- and cell-damaging agents.

These species include both peroxyl and alkoxyl radicals, which have the ability to transfer their free radical nature to biomolecules. e.g., peptides and proteins, low-molecular-mass carbohydrates and polysaccharides, etc., processes giving rise to a series of molecular scissions, cross-linking and polymerisations, and a series of co-oxidation processes; the formation of hydrogen bond-linked adducts of the -OOH functions of HPMs and CHPDs to receptors in proteins and nucleic acids, which, in turn, results in the *in situ* degradation followed by H atom abstraction from, or the addition of lipid-derived radicals to, selected protein localisations; the generation of adducts from the reactions of epoxy-FAs with proteins and other biomolecules; and finally, as outlined in this work, the many reactions of aldehydic LOP fragmentation products with a wide range of biomolecules to form adducts, which can lead to the production of cross-linked biomacromolecules, and fluorescent and browning products, for proteins. An excellent review of these co-oxidation phenomena is available in Schaich (31).

### Thermal Degradation Products Generated During Very High-Temperature Frying Practises

Also of importance are products arising solely from the non-oxidative thermal degradation of lipids, and previous reports have investigated the molecular nature of these in experimental protocols in which thermal stressing was applied under strictly non-oxidative conditions (32). Such reactions include those involved in dehydration, decarboxylation, triacylglycerol hydrolysis, -CH=CH- bond conjugations, dehydrocyclization, dehydrogenation, aromatisation, polymerisation, and -C-C- bond cleavage. The thermal induction and progress of these reaction systems are, of course, highly temperature-dependent, but typically investigations focused on these have been conducted at temperatures of 200–300°C, and therefore the lower- and mid-ranges are relevant to some extreme temperatures applied during certain shallow-frying and Chinese-style cooking practises (33), the latter of which may attain temperatures higher than 250°C.

### Dimerization, Oligomerization, and Polymerization of FA Acylglycerols

Polymeric FA derivatives are highly conjugated diene species, and their time-dependent generation in heated culinary oils during frying processes gives rise to the development of a brown-coloured residue with a resin-like appearance which can often be observed along the sides of the frying vessel, a site where the frying oil, and utensil- or food-derived metal ions come into contact with atmospheric O_2_. These polymers are produced when moisture and air become conglomeratively enmeshed within the oil during the course of frying episodes, and this may arise from the production of a “steam blanket” when food is first introduced into the high-temperature frying oil. Exposure of TAGs to high-temperature frying practises results in the formation of both oxidatively- and non-oxidatively-modified polymeric lipids, for example oxidised TAG species with core epoxy, hydroxy, keto and aldehyde functions (34, 35). As we might expect, the generation of polymerised lipids further accelerate oil deterioration, a process augmenting enhanced viscosity and foam formation, along with the development of undesirable food colourations (36, 37). Polymer formation is also linked to diminished heat transfer and therefore an increased level of oil absorption by fried foods.

These polymeric species, which have an increased polarity, are largely generated through mechanisms involving free radical reactions. Indeed, routes for the formation of such polymers involve the intermediary generation of cyclic fatty acids within a single FA chain, and dimeric FAs produced between two FA moieties, either within the same or between different TAGs. Following this, the polymerisation process is promoted by the cross-linking of dimers with further cyclic FAs and TAGs (38). According to reports, the generation of cyclic adducts is contingent on the unsaturation status of the frying oil, and also the frying temperature. Indeed, the production of cyclic monomers and polymers appears to be linearly-dependent on oil linolenoylglycerol content and frying temperature. However, it is also reported that the linolenic acid content of cooking oils has to be >20% to yield significant quantities of cyclic monomers, although their yield is significantly higher at temperatures ≥200°C.

Notably, the impact of the Diels-Alder reaction in lipid polymer development in thermally-stressed culinary oils has been previously investigated in some detail. Although a number of studies have suggested its importance in polymer generation (38), this feature has been countered by others (39). Notwithstanding, more recent evidence now suggests that Diels-Alder reaction may not play a major role in such thermally-induced polymer formation, and Diels-Alder products produced at levels of <5 mol. % may not be detectable using the now commonly-employed NMR analysis method developed.

### Triacylglycerol (TAG) Hydrolysis Products

In general, the thermally-mediated hydrolysis of TAGs occurs within the bulk oil phase and not the water-oil interface (40). Although the steam blanket arising from both air and food moisture when foods are first introduced into high-temperature oil matrices subsides during the frying process, air captured therein gives rise to a series of hydrolysis reactions, in addition to peroxidation and polymerization processes. Primarily, cleavage of the ester linkage of TAGs results in the production of mono- and diacylglycerols, and free FAs (FFAs). The oil content of such FFAs accelerates with increasing number of frying cycles (41), and therefore these marker molecules, and their proportionate conductivities, are hence employed for the quality monitoring of frying oils. Major hydrolysis products produced formed are monoacylglycerols, diacylglycerols, FFAs and free glycerol. FFAs themselves can undergo oxidatively- and thermally-induced polymerization processes, which then give rise to the formation of TAG dimers, trimers, oligomers, and polymers.

Interestingly, the higher the level of short and unsaturated fatty acid acyl chains in oils, the greater the amount of hydrolysis observed, with long and saturated fatty acid chain-rich oils being more resistant to this process. This is attributable to shorter and unsaturated fatty acid chains being more soluble in aqueous media (32, 40). However, regular replacement of used with fresh oils, and not the frying period, suppresses the hydrolysis process, and therefore also suppresses polar compound generation (42). As expected, the cleansing of fryers and frying utensils with sodium hydroxide and other alkaline media accelerates oil hydrolysis.

Mono- and diacylglycerol hydrolysis products are produced throughout the early phases of frying practises in view of the breakage of steam bubbles and generation of a steam blanket over the oil surface, a process resulting from the high interfacial tension of the frying system (43, 44). However, since the steam blanket diminishes with continued frying, the oxidation of UFAs is then expedited.

## Dependences of Aldehydic LOP Production Rates and Generated Concentrations on the FA Compositions of Culinary Frying Oils Exposed to Standard Frying Practises

A wealth of previous reports has demonstrated that, when standardised to frying type, frying vessel size, and frying temperature and duration, the molecular nature and concentrations of aldehydes detectable in culinary oil samples exposed to high-temperature frying practises is critically dependent upon their FA acylglycerol composition, with PUFA-rich oils such as corn oil engendering much higher levels than MUFA-rich ones (e.g., olive oil), as noted above. Those with only low UFA levels such as coconut oil generate little or no aldehydes, whereas oils with relatively high contents of ω-3 FAs generate patterns of aldehydes which are both MS- and ^1^H NMR-distinguishable from those arising from the peroxidation of ω-6 FAs (45–49). Although this is indeed a generalised conclusion, it is quite correct, and these observations originally made in out laboratory have been largely ratified and validated by those made in many other laboratories globally (46). These data are fully consistent with the known rates of peroxidation which are in the order linolenic acid > linoleic acid >>> oleic acid >>>>>> stearic acid (7–9). Furthermore, the rate of degradation of CHPDs or HPMs to lower-molecular-mass fragmentation products such as aldehydes also decreases with decreasing FA saturation status; specifically, it is in the linolenoyl- > linoleoyl- >>> oleoylglycerols order. Hence, the higher levels of thermally-induced aldehyde generation in frying oils are found in PUFA-rich oils, with those containing large amounts of ω-3 FAs such as marine oils producing even greater concentrations.

One of our group's previous studies (47) compared the time-dependencies of aldehydic LOP generation for sunflower, corn, canola, extra-virgin olive and a newly-developed MUFA-rich algae frying oil [MUFA content 91.2% (w/w)]; the molar [MUFA]:[PUFA] % content ratios of these products were 0.46, 0.38, 2.26, 8.23 and 21.71, respectively. This result is entirely typical of those achieved by other laboratories in that high PUFA/low MUFA content oils generate much higher levels of aldehydes and at a more rapid rate than those with high MUFA/low PUFA contents (49, 50). Indeed, at the extreme 90 min laboratory-simulated shallow frying episode time period, the total mean ± SEM saturated and α,β-unsaturated aldehyde contents of the oils evaluated were 3.56 ± 0.42 and 17.22 ± 0.60 (sunflower oil); 3.13 ± 0.25 and 15.67 ± 0.47 (corn oil); 2.32 ± 0.04 and 11.53 ± 0.14 (canola oil); 2.48 ± 0.09 and 10.62 ± 0.38 (extra-virgin olive oil); and 0.55 ± 0.09 and 4.78 ± 0.80 (MUFA-rich algae frying oil), respectively. Hence, for all oils evaluated, a surprisingly consistent 81–90% of the total aldehydes detected comprised the more toxic α,β-unsaturated classifications for all oils investigated. Similarly, a further investigation conducted by Almosehy et al. (51) found that the peroxidative stability order of culinary oils was moringa oil > extra-virgin olive oil > apricot kernel oil > sunflower oil, and for these experiments ^1^H NMR analysis results were found to compare favourably with those derived from application of the Rancimat thermo-oxidative method.

As noted above, also of much importance are dependencies of the patterns of aldehydic LOPs generated from differential FA substrates. For example, PUFAs in general form a wide realm of such aldehydes from the peroxidation process, including alka-2,4-dienals with two -HC=CH- double bond units, along with a range of substituted (*E*)-2-alkenals such as 4-hydroperoxy-, 4-hydroxy- and 4,5-epoxy- adducts, whereas MUFA peroxidation yields only limited numbers of aldehyde classes, most predominantly *n*-alkanals and (*E*)-2-alkenals (47). Additionally, selected types of aldehydes may arise as tertiary LOPs from the thermal oxidation or isomerism of secondary aldehydic LOPs, for example acetaldehyde from the degradation of malondialdehyde (MDA), isomeric alka-2,4-dienals, or 2,3- or 4,5-epoxyaldehydes during high temperature frying processes, and (*cis,trans*-)-alka-2,4-dienals from their corresponding (*trans,trans*)- isomers, as reviewed in Moumtaz et al. (47).

### Considerations of the Volatilities of Aldehydic LOPs: Relevance to Their Contents in Frying Oil and Fried Food Matrices, Their Adverse Inhalation by Chefs and Restaurant Workers, and Frying Temperature

In view of its relatively high volatility, and marginally greater chemical reactivity with physiological substrates such as free amino acids, glutathione (GSH), proteins and DNA, acrolein may be considered atypical of higher homologue 2-alkenal species. However, other α,β-monounsaturated aldehydes besides acrolein also have a high level of volatility, for example *trans*-2-hexenal, *trans*-2-pentenal and methacrolein have boiling points (b.pts) of 47, 80–81 and 69°C, whereas that of acrolein itself is 53°C. Additionally, the saturated aldehydic LOPs formaldehyde, acetaldehyde and propanal have b.pts of only −19, 20, and 49°C. A full list of the b.pts of a wide range of aldehydic LOPs, along with their original triacylglycerol/FA sources is provided in Table 1.

**Table 1:** Boiling points (b.pts) of aldehydic LOPs (at 760 mm Hg) liberated from UFAs.

**Aldehyde**	**B.pt (**°**C)**	**Acylglycerol chain or alternative TAG sources**
Formaldehyde	−19	Decomposition of MDA
Acetaldehyde	20	Alka-2,4-dienal decomposition
Propanal	49	Ln
*n*-Pentanal	102–103	L
*n*-Hexanal	131	L/Alka-2,4-dienal decomposition
*n*-Octanal	173	L/O
*n*-Nonanal	79–81	O
*n*-Decanal	213	O
Acrolein	53	Ln/Glycerol Backbone/Alka-2,4-dienal decomposition
(*E*)-2-Heptenal^*^	166	Alka-2,4-dienal decomposion
(*E*)-2-Octenal	84–86	L/Alka-2,4-dienal decomposition
(*E*)-2-Nonenal	205	L
(*E*)-2-Decenal	230	O
(*E*)-2-Undecenal	234	O
(*E,E*)-Hepta-2,4-Dienal	177	Ln
(*E,E*)-Deca-2,4-Dienal	115	L
4-Hydroxy-(*E*)-2-hexenal	233.5 ± 23	Ln
4-Hydroxy-(*E*)-2-nonenal	276 ± 23	L
Malondialdehyde	108 ± 23	Ln

*Their triacylglycerol sources are also listed*.

*L, Linoleic acid; Ln, Linolenic acid; O, oleic acid acylglycerol chains; MDA, malondialdehyde; E, trans-configuration*.

* *2-heptenal isomers are derived from alka-2,4-dienal decomposition, along with acetaldehyde, hexanal, acrolein, butenal, 2-heptenal, 2-octenal, benzaldehyde, glyoxal, and trans-2-buten-1,4-dial (52)*.

These data are clearly of much relevance to their retention in fried food matrices, but perhaps more importantly by their direct inhalation by subjects domestically conducting frying or even wok-cooking episodes, or those employed in poorly-ventilated restaurants who are continuously exposed to cooking oil fumes. Indeed, we may surmise that the lower the b.pt, the lower the level of aldehydes retained in frying oil and fried food media, and vice-versa. whereas conversely higher levels of such low b.pt aldehydes will be found in cooking oil fumes. However, there are, of course, many other physicochemical considerations which influence the relative levels of each aldehydic LOP in these distinct environments.

As expected, the vaporisation of aldehydic LOPs increases with increasing temperature, as does the rate and extent of peroxidation in general, and therefore it is anticipated that a frying oil temperature of 180°C will yield higher levels of these toxins in both the cooking oil itself and fumes arising therefrom than those at a lower frying temperature of say 150°C. Indeed, although it is now generally accepted that the best frying temperature should lie within the 160–180°C range for most frying operations, temperatures as low as *ca*. 120°C may be employed for selected frying media, e.g. vegetable shortening oils. Moreover, some countries have now set frying temperature limits of 160°C, a valuable health-wise strategy since this may be expected to reduce the levels of dietary LOPs available for human consumption.

## Dietary Consumption of Fried Foods by Humans in the Western World, With Special Reference to French Fries

Although the mean daily intake of potatoes as French fries in the UK is 20 g, plus 10 g for the contribution of oven chips, as noted in Gibson and Kurilich (53), in Moumtaz et al. (47) we clearly specified that the concentrations of aldehyde toxins were those estimated in fast-food restaurant portion sizes of either 71, 154, or 400 g so that readers could clearly appreciate their contents in such a context. French fries are certainly one consideration, albeit a major one, but of course there are many other types of peroxidised frying oil-laden fried foods available for human consumption, including burgers, meat patties. fried chicken, eggs and donuts, etc., most especially if frying oils of high PUFA content have been used for their preparation. Moreover, we have shown that such alternative fried foods and fried food products such as fried chicken and pork sausages (47) also contain patterns of aldehydic and further hazardous lipid oxidation products (LOPs), the latter including epoxy-fatty acid toxins such as 9,10-epoxy-12-octadecenoate (leukotoxin) and its more potent corresponding diol (23, 47, 54), which have leukocyte degeneration and necrotizing properties (23), and are involved in the pathogenesis of multiple organ failure and breast cancer (54); they can also interfere with the reproductive functions of rats (23).

In 2013, Gibson and Kurilich (53) explored the nutritional contribution of potatoes and potato products toward the UK diet from a secondary analysis of 4-day dietary records arising from the National Diet and Nutrition Survey (2008–2011)^1^. For this purpose, they also differentiated between fried and oven cooked chips. Of 92% of survey respondents consuming potatoes during the 4-day trial, 41% were found to consume fried chips, and 27% consumed oven chips. The mean potato consumption level was 85 g/day per capita, of which fried and oven chips constituted means of 20 and 10 g/day, respectively. Moreover, for French fries, mean serving sizes were 189 g for adults, 138 g for teenagers, and 94 g for 4–10-year old children; corresponding values for oven chips were somewhat lower. Interestingly, potatoes only contributed 4% of the total human consumption level of SFAs. Additionally, at the time of publication of this report, total potato consumption level in the UK was diminishing at a rate of 1–2% per annum, and this was linked to increases in the consumption of alternative starchy staple foods such as rice and pasta.

A further important point is that a daily 60 g value of potato chip consumption in the UK, i.e., double the estimated mean value above for combined fried and oven chip consumptions, is sometimes described as a “worst-case scenario.” However, how is this plausible when it is well-known to be possible for at least some humans to consume higher or much higher average daily amounts of this fried food? Estimates for potato and French fry consumption in the USA are averages of 137 and 37 g/day, and hence this vegetable is consumed more so than in the UK, both overall and as French fries.

According to the National Diet and Nutrition Survey (NDNS), UK males and females consume averages of 9 and 6 g of crisps and potato snacks, savoury or otherwise, per day (55). This observation is also of much importance, since quite high levels of the most predominant aldehydic LOPs are also readily detectable in these frequently-consumed products (56, 57). So, that adds another 8.6 g onto the above average UK French fry consumption rate of 30 g/day.

Although not strictly relevant to UK or US diets, a recent investigation focused on the consumption of fried foods in a very large Spanish cohort found that a mean daily quantity of 138 g of fried food was consumed (58), and this figure included 34g/day fish, 31 g/day meat, 30 g/day potatoes, and 15 g/day eggs. Therefore, the above 138 g/day mean value is more than 4-fold greater than the above mean 30 g/day estimate for French fries and oven-cooked potato chips in the UK; it seems that we do not live on French fries alone. Interestingly, 62% of participants in this study used peroxidation-resistant, monounsaturated fatty acid (MUFA)-rich olive oil for frying purposes, whilst the remainder used sunflower or alternative vegetable-derived oils—this may at least partially explain why no associations between fried food consumption and coronary heart disease, nor all-cause mortality risk, were found in this study.

In Spain, both sunflower and olive oils are the predominant products employed for culinary frying episodes. Indeed, Spaniards mainly consume olive oil for this purpose (average annual consumption per capita of 4.01 litres in 2019), whereas sunflower oil was Spain's second most popular frying oil in 2019, with consumption levels of 3.61 L per capita in 2019^2^. However, in addition to refined olive oil products, for 2019 consumption values of 0.71 and 2.99 L per capita were recorded for virgin and extra-virgin olive oils in that country. Although these virgin products, usually with lower or much lower smoke-points, are almost exclusively used for dressings, etc. as part of a Mediterranean diet, at least a small proportion of them are presumably intentionally or mistakenly also employed for high-temperature frying purposes.

## Estimated Dietary Intakes of Acrolein and Other Aldehydes

### Overview of Dietary Aldehyde Intake in Humans

Acrolein represents the simplest, lowest homologue 2-alkenal class of α,β-unsaturated aldehydes, and is conceivably one of the most toxic of these LOPs. One estimate for dietary acrolein intake (only 70 μg/day) arises from only limited data available as far back as 2001 (59). Under this EU Risk Assessment Report document's section 4.1.1.3.4 heading “***Exposure through food and*** ***beverages, and natural sources***,” references utilised for this estimate are dated from 1991 to 1996, and the authors themselves state that “It is very difficult to estimate the human dietary intake of acrolein from foodstuffs and commodity articles.” This author certainly does not disagree with them!

With regard to the risk report documented in (61), a more recent (2008) and perhaps more reliable estimate of mean dietary acrolein intake from a total of only 8 foods is as much as 2.35 mg/day (equivalent to 34 μg/kg for a 70 kg body weight (BW) human) (60), and this value is 33-fold greater than the above likely underestimate. Moreover, the maximal level of this aldehyde's dietary exposure was estimated as 5 mg/day. Full details of these estimates are provided in Table 2.

**Table 2:** Acrolein consumption from the 8 foods listed is 2.35 mg daily.

**Food**	**Acrolein content**	**Daily consumption**	
		**Food**	**Acrolein**
Cheese	1.0 mg/kg	40 g	40 μg
Donuts	0.9 mg/kg	400 g	360 μg
Codfish fillet	0.1 mg/kg	100 g	10 μg
Wine	3.8 mg/l	400 ml	1,520 μg
Fruits	0.05 mg/kg	300 gm	15 μg
Vegetables	0.50 mg/kg	500 gm	250μg
Potatoes	0.60 mg/kg	250 gm	150 μg
Oil	0.200 mg/kg	50 gm	10 μg

*Acrolein has been detected in 35 food substances and therefore the maximal estimated consumption of acrolein via food is likely to be 5.0 mg per day or 0.1 mg/kg/day [for a 50 kg BW human]. Smoking cigarettes, which generate 50–100 μg of acrolein per cigarette, can result in additional exposure of up to 0.2 mg/kg/day [this Table was reproduced from Wang et al. (60) with permission]*.

#### Daily Consumption of Acrolein From Food

Similarly, human exposure levels for total aldehydes have been estimated to be as much as 7 mg/kg/day, with 5 mg/kg/day representing the more reactive and toxic α,β-unsaturated class (5). Astonishingly, these values represent total mean values of 490 and 350 mg/day, respectively, for an average 70 kg BW human (60). Although this author personally finds these estimates a little daunting, such estimated intakes clearly substantially exceed those estimated from the use of aldehydes as food flavourants, or alternative previous authoritative environmental and/or dietary estimates for them.

Although not an ideal situation, in Moumtaz et al. (47), toxicological intake estimates were related back to acrolein since it is one of the very few unsaturated aldehydes with documented authoritative Acceptable Daily Intake/Tolerable Daily Intake (ADI/TDI) values available. Acrolein is generated during the frying of culinary oils which contain significant levels of linolenoylglycerols (e.g., soybean or canola oils), or from oxidation of the glycerol backbone of oil triacylglycerols (TAGs) in general (61). In view of their high omega-3 fatty acid contents, we can readily detect and quantify acrolein in thermally-stressed or peroxidised marine oils (56).

### Human Aldehyde Intake From Fried Food Sources

Total culinary frying oil concentrations of saturated and α,β-unsaturated aldehydes in sunflower oil heated at 180°C for a 20 min duration were found to be 0.88 and 3.90 millimoles per mole of total fatty acid (mmol./mol. FA), equivalent to *ca*. 3 and 13 mmol./kg of oil, respectively (26). Corresponding values for corn oil were very similar (0.87 and 3.56 mmol./mol. FA, respectively), whereas those for a novel algae-derived frying oil containing 92% (w/w) trioleoylglycerols were only 0.08 and 0.51 mmol./mol. FA, respectively (47). Therefore, on consideration of the most predominant *n*-alkanal and *trans*-2-alkenal aldehydes arising from the peroxidation of linoleoylglycerols present in PUFA-rich sunflower and corn oils, i.e., *n*-hexanal and *trans*-2-octenal, these levels would be equivalent to no less than 300 and >1,500 ppm, respectively. Similarly, if the unsaturated aldehydes were exclusively of the alka-(*trans,trans*)-2,4-dienal classification, i.e., deca-(*trans,trans*)-2,4-dienal from the fragmentation of linoleoylglycerol hydroperoxide sources, this level would be very nearly 2,000 ppm.

Hypothetically, if the frying oil concentrations of total saturated and α,β-unsaturated aldehydes (the latter including contributions from acrolein) were 3.0 and 13.0 mmol./kg oil, respectively, and a 100 g portion of UK potato chips or French fries contained, say. 15% (w/w) of such an oil, then without considering any chemical consumption of them by reactions with potato proteins, free amino acids and/or carbohydrates, their contents in this fried food portion would be as much as 0.45 and 1.95 mmol./kg, respectively (corresponding to 39 and 246 ppm of *n*-hexanal and *trans*-2-octenal, the most predominant *n*-alkanal and *trans*-2-alkenal species arising from linoleoylglycerol peroxidation, respectively), i.e., ~4 and 23 mg of these aldehydes per serving. Similarly, if these α,β-unsaturated aldehydes exclusively comprised deca-(*trans,trans*)-2,4-dienal, the major alka-(*trans,trans*)-2,4-dienal derived from linoleoylglycerol peroxidation, the potato chip content of it would be as high as 28 mg per 100 g portion (297 ppm), albeit again without allowing for their chemical reactivity with potato biomolecules. Nevertheless, the total lipid content of potato chip samples collected from fast-food restaurants can vary from <1 to *ca*. 35% (w/w) (30), and therefore the above mg and ppm aldehyde contents are critically dependent on their lipid contents, but these values may reach levels which are ~ 2-fold greater than that estimated above.

However, analytical estimates of total *n*-alkanals, *trans*-2-alkenals and alka-(*trans,trans*)-2,4-dienals in potato chip samples were found to be 121 ± 33, 157 ± 43, and 126 ± 25 μmol./kg using ^1^H NMR analysis (47). One further reversed-phase HPLC estimate for the maximal concentration of deca-(*trans,trans*)-2,4-dienal in French fries deep-fried in sunflower oil in a domestic deep-frying apparatus at 170°C was *ca*. 65 μmol./kg (10–11 ppm) (62), but obviously this value is for this aldehyde alone, and not others present within the alka-(*trans,trans*)-2,4-dienal classification.

Hence, in Moumtaz et al. (47), the levels reported, which correspond to total *n*-alkanal and α,β-unsaturated aldehyde concentrations of *ca*. 0.12 and 0.28 mmol./kg, respectively, are actually lower than those estimated for them in the above hypothetical model, which assumes that 15% (w/w) of the potato chip product mass comprises thermally-stressed culinary oil infiltrated therein during the frying process; this is accountable by the chemical consumption of such aldehydes via reactions with French fry potato biomolecules, as outlined below. ^1^H NMR-determined lipid contents of the fried food products were found to be ~10 and 15% (w/w) for two commercially-differing retail restaurants (63), and therefore very comparable to the above 15% (w/w) estimate. The lower aldehyde contents reported in Moumtaz et al. (47) will, of course, be critically dependent on a wide range of factors, for example frying temperatures and fried food exposure times, potential recycling of reused oils, frying type, and frying oil MUFA, PUFA and perhaps antioxidant contents, together with their reactivities with potato chip biomolecules, etc. Mean MUFA and PUFA contents of the frying oils tested in Moumtaz et al. (47) were 38 and 35% (w/w), respectively (ranges 25–54 and 8–55%, respectively), whereas those for the oils evaluated in Boskou et al. (62) were ~60 and 30% (w/w) for the two restaurants involved.

Typical concentration ranges for each of the 3 major aldehydic LOPs (*n*-alkanals, *trans*-2-alkenals and *trans,trans*-alka-2,4-dienals) in non-French fry fried foods purchased from fast-food/take-out retail outlets in the UK are 10–50 and 100–420 μmol./kg for the meat and outer batter covering portions, respectively, of battered fried chicken (*n*-alkanals and *trans*-2-alkenals only for meat); 20–100 μmol./kg for fried sausage (*n*-alkanals only); 60–150 μmol./kg for fried onions; and 180–600 μmol./kg for fried potato slices. For Chinese-style take-out products, these ranges are 10–50 μmol./kg for egg-fried rice; 30–120 μmol./kg for the meat portion only of battered chicken balls; and 50–140 μmol./kg for prawn crackers (our previously unreported high-resolution ^1^H NMR analysis data). Such μmol./kg aldehyde contents may be adjusted so that the μg amounts of them available for human consumption per 71, 100, 154, or 400 g portion can be calculated proportionately.

As with French fry or UK potato chip samples, these concentration ranges are critically dependent on the unsaturation status of the frying oils used, and the amount of them absorbed by the foods specified during frying, together with a variety of other factors such as frying conditions, durations, temperatures, and oil reuse. However, these ranges are similar to those found for French fry samples (47). As we might expect, the batter coverings of selected fried meats and fish always contain significantly higher levels of aldehydic LOPs than the meat portion itself, and for battered chicken samples, this difference was found to be ~5–10-fold greater. Hence, it appears that such batter envelopes provide protection of meat products against the infiltration of frying oil-borne aldehyde toxins during high-temperature frying episodes.

In addition to their determined concentrations, one important notable feature of these reported datasets is differences between the “patterns” of aldehydes in the potato chip products and that of the oils in which they were fried. Indeed, we find that although PUFA-rich frying oils contain molar concentration levels of ≥75% α,β-unsaturated and ≤25% saturated aldehydes, respectively (the former comprising variable but often similar quantities of *trans*-2-alkenals and *trans,trans*-alka-2,4-dienals), the molecular aldehyde composition of French fries always contains higher or much higher proportions of *n*-alkanals, for example nearly 40 mean molar % in Boskou et al. (63), and *ca*. 31 mean molar % in Moumtaz et al. (47). This observation is likely to arise from the higher chemical reactivities of α,β-unsaturated aldehydes with fried food biomolecules to form Michael addition or Schiff base adducts (from reactions with amino acids or proteins), or hemiacetal and subsequently acetal products (from reactions with starch and other carbohydrates), than those of *n*-alkanals, which is now a commonly recognised phenomenon in this research area (56); *n*-alkanals can only form Schiff base products through the reactivity of its terminal-CHO function. Indeed, only the α,β-unsaturated aldehydes generate Michael addition products through nucleophilic attack of thiolate or amine functions at their electrophilic-C3 sites (64). Hence, reactivity differences such as these should, at least in principle, result in proportionately higher levels of saturated aldehydes in fried foods, although this will also be largely dependent on the stability of aldehyde-reactive biomolecules when exposed to high frying temperatures, and also perhaps the aldehyde-scavenging properties of products arising from their thermal degradation.

Hence, it is expected that the availability of such aldehyde-consuming agents will be depleted somewhat, perhaps even substantially so, when exposed to high-temperature frying episodes. However, the toxicological significance of these chemical transformations remains a subject of conjecture. Firstly, it should be noted that the levels of aldehydes determined in fried food products by our group (26, 37), and those by Csallany et al. (65) focused on 4-hydroxy-*trans*-2-nonenal only, are restricted to those of the “free,” i.e., unreacted, aldehydes, and such specific analyses did not consider or include any which have been chemically consumed and hence derivatised by reactive biomolecules. Hence, the concentrations provided represent the free aldehydes that were uptaken from thermally-stressed frying oils by French fry samples during the frying processes featured. However, as reported by Esterbauer et al. (64) for Michael addition products of α,β-unsaturated aldehydes, such chemically-modified aldehydes may serve as latent sources of them *in vivo* in view of the reversibility of such reactions, as indeed they might following their human intake. Such phenomena may potentiate or prolong the bioactivities and adverse health effects of dietary sources of these secondary LOPs.

However, in 2016, Wang et al. (66) investigated the kinetics and mechanisms of aldehyde production in thermally-stressed cooking oils, using an LC–MS analytical technique coupled with, principal component analysis (PCA) and hierarchical clustering analysis (HCA) to explore patterns of these LOPs. They found that the aldehydes significantly apportioned to the time-dependent distinction of heated soybean oil samples in a PCA model were segregated into three clusterings with HCA according to rates of generation and FA sources. Increases observed in 4-HNE concentration, and the ratio of two of these aldehyde cluster levels in this oil were strongly correlated with heating period. Of especial interest, multivariate (MV) chemometric and quantitative analysis of replicate samples of extracts of five French fry samples fried in either soybean, corn, and canola oils provided further evidence for relationships between aldehyde profiles and their FA substrates, and also demonstrated that *n*-pentanal, *n*-hexanal and acrolein levels, and the above cluster ratio in these extracts were not dissimilar to these indices in corresponding frying oil media, whereas those observed for all other unsaturated aldehydes, were generally found to be one order of magnitude higher in the oil samples analysed. The authors concluded that specific aldehydes or clusterings of such aldehydes may serve as valuable markers of lipid peroxidation status for frying oils and/or fried foods. Our estimated range (median) concentrations of the oil extracted from French fry aldehydes analysed in this communication are: *n*-pentanal 1-3 mg/L (25 μmol/kg); *n*-hexanal 3-14 mg/L (95 μmol./kg); acrolein 3-10 mg/L (131 μmol./kg); 2-heptenal 0-9 mg/L (43 μmol./kg); 2-octenal 0-5 mg/L (22 μmol./kg); 2-decenal 0-18 mg/L (64 μmol./kg); 2-undecenal 0-20 mg/L (64 μmol,/kg); 2,4-heptadienal 0-7 mg/L (35 μmol./kg); 2,4-decadienal 0-50 mg/L (178 μmol./kg); 2,4-undecadienal 0-20 mg/L (9.0 μmol/kg); HNE 0-4 mg/L (13 μmol./kg). A uniform weight per mL cooking oil value of 0.921 g/mL was used for converting these aldehyde concentrations to μmol./kg units. As might be expected, the major linoleoylglycerol precursor-derived aldehyde 2,4-decadienal had the highest concentration present in these extracts. Notably, the LC-MS technique used for these analyses was unable to distinguish between geometric isomers of the 2-alkenals and 2,4-dienal species explored, whereas the ^1^H NMR strategy employed in our studies can readily detect, track and quantitate both *cis*- and *trans*-alkenals, and *cis,trans*- and *trans,trans*-alka-2,4-dienals separately.

Of particular interest to the current study, the concentrations of the total FA-normalised saturated aldehydes *n*-pentanal and *n*-hexanal were found to be comparable to those found in the frying oils used themselves, an observation similar to that found in our previously reported investigations, as noted above. Again, one plausible explanation for this is the higher reactivities of α,β-unsaturated aldehydes with potato or other frying item biomolecules such as amino acids or carbohydrates. However, the ability of potato tuber constituents such as trace levels of transition metal ions, or other components, to catalytically promote the degradation of unsaturated aldehydes to saturated derivatives, or saturated thermal degradation products, remains a possibility.

**Figure 1:**
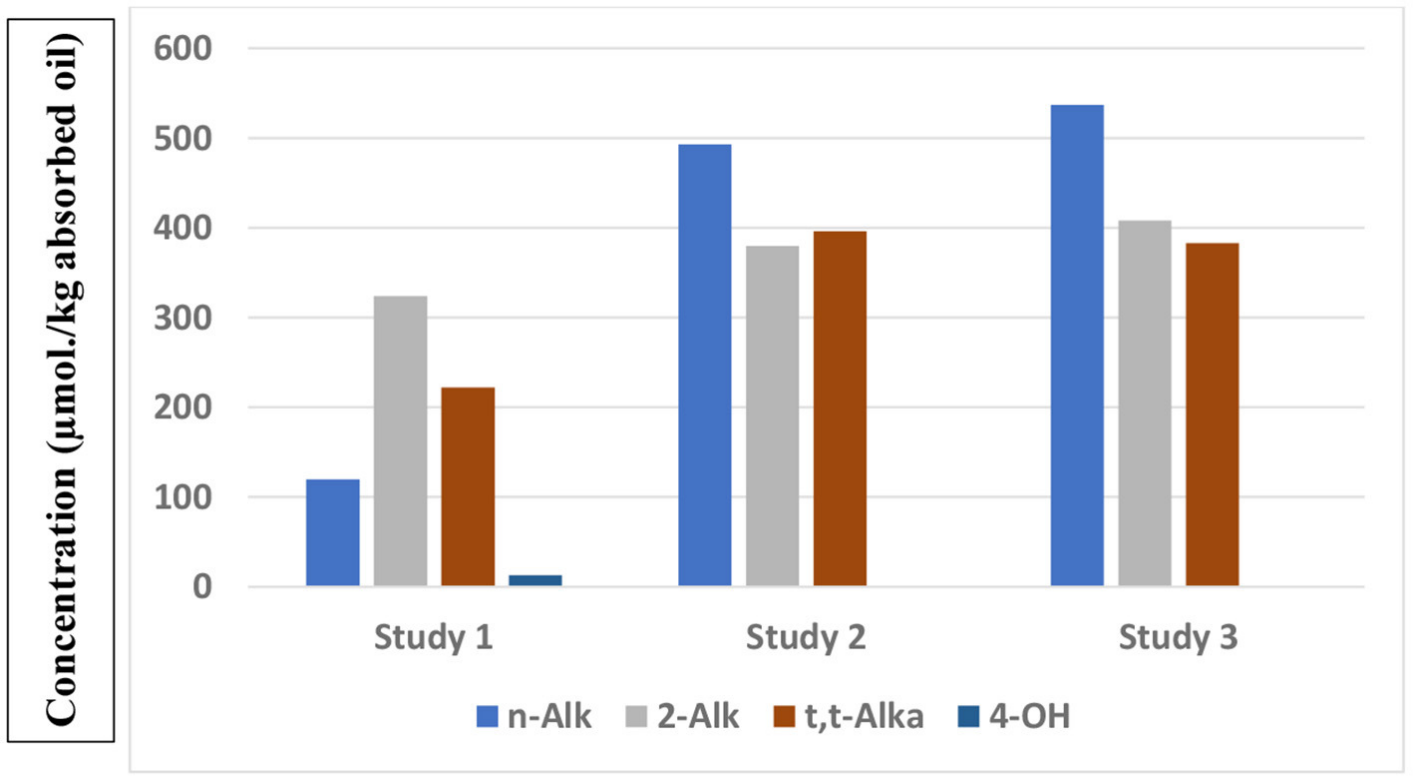
Total mean μmol./kg absorbed oil contents of different classes of aldehydic LOPs present in French fry or potato chip samples purchased from fast-food restaurants, as reported in three separate studies (studies 1, 2, and 3), with *n* = 10, 12, and 44 French fry or potato chip samples tested, as reported in Csallany et al. (66), Moumtaz et al. (47), Grootveld et al. (57), respectively). Study 1 investigated US French fry samples, whereas Studies 2 and 3 analysed UK potato chip samples. n-Alk, 2-Alk, t,t-Alka and 4-OH represent *n*-alkanals, *trans*-2-alkenals, *trans,trans*-alka-2,4-dienals and 4-hydroxy-*trans*-2-alkenals respectively.

Results from a total of three studies which have reported the concentrations of a series of aldehydes present in French fry or potato chip samples collected from fast-food restaurants are shown in Figure 1. Whereas, those available in studies 2 and 3 are quite similar, with the exception of *trans*-2-alkenals, those found in Study 1 were notably smaller. However, this observation is likely to be ascribable to a limitation on the number of *n*-alkanals analysed in Study 1 (*n*-pentanal and -hexanal only). Conversely, ^1^H NMR analysis determines the total levels of this class of aldehyde, although it can also distinguish between medium/long-chain *n*-alkanal homologues and the smaller ones such as propanal and *n*-butanal (47). Data from Studies 2 and 3 again confirm that potato chip samples contain a much higher proportion of saturated:unsaturated aldehyde content ratio (~2:3) than that found in the corresponding thermally-stressed oils [*ca*. 1:3 (47)]. From the results of Studies 2 and 3, assuming a total lipid content of 15% (w/w), estimated potato chip concentrations of a typical 154 g “large” serving of potato chips are 12, 9 and 9 μmol. for *n*-alkanals, *trans*-2-alkenals and *trans,trans*-alka-2,4-dienals, respectively.

### Partial and Canonical Correlation Analysis to Investigate Relationships Between the Patterns of Aldehydic LOPs in Potato Chips and Corresponding Frying Oils

Of particular interest are the potential relationships between potato chip or French fry contents of aldehydic LOPs and those of the frying oils employed to fry them. To explore this further, partial correlation coefficients (and their corresponding two-tailed significance (*p*) values) were computed between a multivariate (MV) dataset containing the potato chip concentrations of aldehydes and those of their corresponding frying (sunflower) oil ones as a function of frying duration time-point (Table 3). These data were obtained from an experimental regimen described in Moumtaz et al. (47), in which eight batches of hand-cut potato chips (length 87.00 ± 1.15 and width 12.70 ± 0.36 mm (mean ± SEM)), respectively, were sequentially fried 8 times using sunflower oil. For this purpose, we used a domestic model deep-frying facility, which was filled with 3.00 litres of oil in accordance with the manufacturer's instructions. 400±10 g masses of potato chips were then deep-fried at a temperature of 170°C for a 10.0 min duration. The oil was then reused 7 times for repeated 10 min potato chip frying episodes.

**Table 3:** Partial correlation coefficient matrix of values observed between the concentrations of the most predominant aldehydic LOP classes present in sunflower oil and those in UK-style potato chip portions sequentially fried for a 10 min period in this medium with reuse of the oil for up to 8 deep-frying sessions in a domestic deep-frying device at a temperature of 170°C.

		**Chips**			**Oil**		
		**(*t*)-2-Alk**	**(*t,t*)-Alka**	***n*-Alk**	**(*t*)-2-Alk**	**(*t,t*)-Alka**	***n*-Alk**
Chips	(*t*)-2-Alk	1	−0.20	0.20	−0.41	0.60^*^	0.11
	(*t,t*)-Alka	−0.20	1	0.40	0.07	0.51	−0.52
	*n*-Alk	0.20	0.40	1	0.25	−0.24	−0.18
Oil	(*t*)-2-Alk	−0.41	0.07	0.25	1	0.76^**^	0.77^**^
	(*t,t*)-Alka	0.60^*^	0.51	−0.24	0.76^**^	1	−0.25
	*n*-Alk	0.11	−0.52	0.18	0.77^**^	−0.25	1

(E)-2-Alk, (E,E)-Alka and n-Alk represent trans-2-alkenals, trans,trans-alka-2,4-dienals and n-alkanals, respectively. Asterisks indicate the statistical significance of these values (^*^p <0.05;

** *p < 0.01). The raw oil and potato chip concentration dataset was generated in the study described in Moumtaz et al. (47)*.

Although a number of negative correlations were observed, such as those between potato chip and oil *trans*-2-alkenal levels (*r* = −0.41), and that between potato chip (*trans,trans*)-alka-2,4-dienal and oil *n*-alkanal concentrations (*r* = −0.52), the only significant partial correlations found were positive ones observed between the oil contents of both (*trans,trans*)-alka-2,4 dienals and *n*-alkanals with oil *trans*-2-alkenals, and also that between frying oil *trans,trans*-alka-2,4-dienals and potato chip *trans*-2-alkenals.

These observations provide evidence for significant partial correlations between the frying oil aldehyde concentrations (although not so between *trans,trans*-alka-2,4-dienals and *n*-alkanals), as may be expected in view of their production levels being stimulated by increasing frying reuse cycle durations. However, a significant partial correlation was also found between oil *trans,trans*-alka-2,4-dienal and potato chip *trans*-2-alkenal levels, and this may indicate that when making contact with, or uptaken by the water-containing fried food matrix, the former aldehydes degrade to structurally-simpler alkenal adducts.

Canonical correlation analysis (CCorA) was also performed in order to explore inter-relationships between two series of aldehydic LOP datasets, the first comprising the sunflower oil concentrations of *n*-alkanals, *trans*-2-alkenals and *trans,trans*-alka-2,4-dienals, along with the 10.0 min frying session episode number (0–8), the second (“dependent”) one consisting of the potato chip concentrations of each of these aldehydes only. The dataset was autoscaled, and subsequently CCorA was conducted to investigate inter-relationships between them, together with the dimensionality of such canonical correlations; these canonical dimensions represent latent variables which are analogous to factors in factor analysis, although such canonical variates serve to maximise correlations between the two sets of variables investigated.

**Figure 2:**
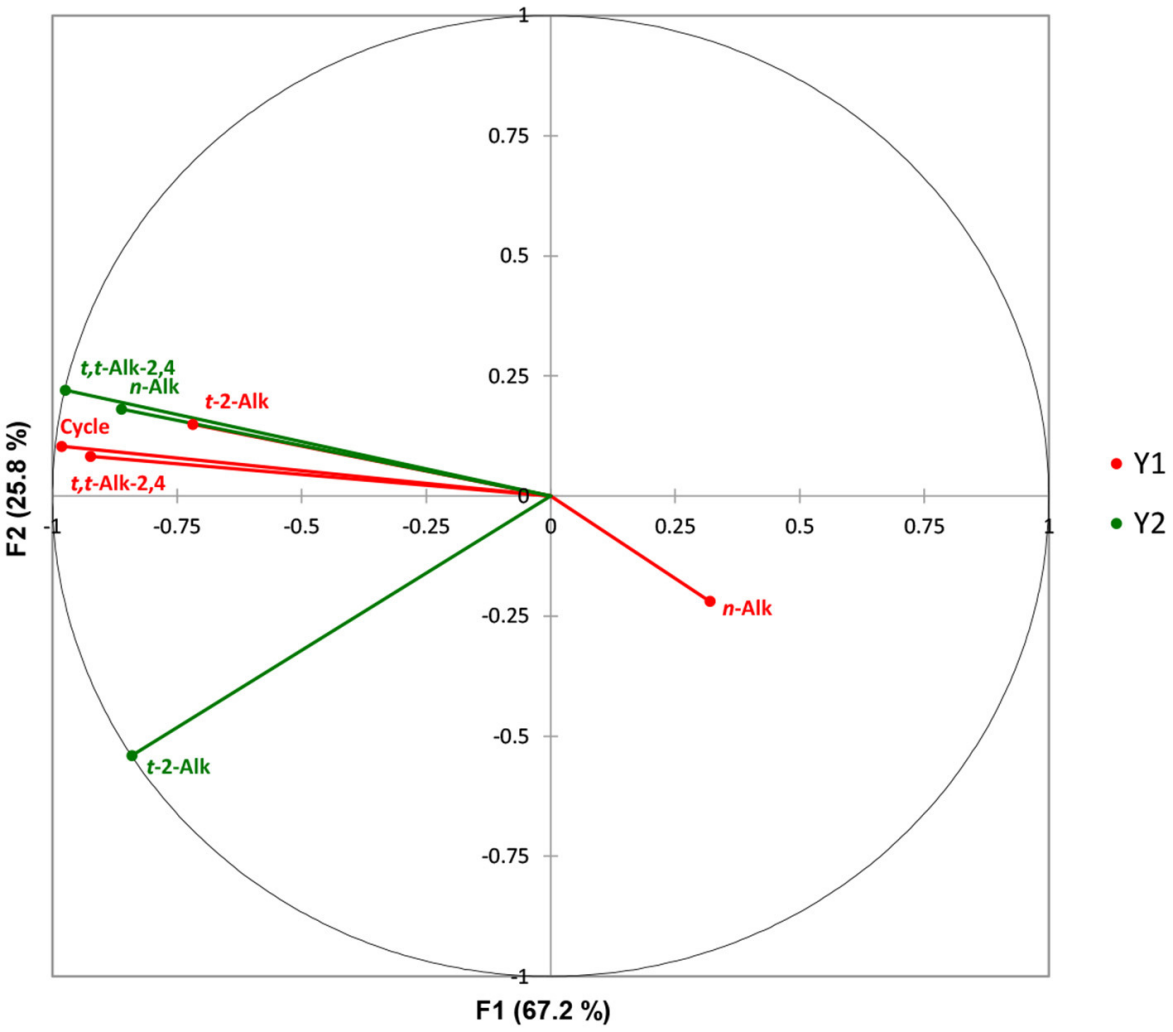
Canonical correlation plot of the second vs. the first factor involved in the CCorA analysis. The frying reuse codes ranged from 0 to 8, where 0 represented unheated oil, and 1–8 those exposed to increasing sequential 10.0 min frying episodes at a thermostatically-maintained temperature of 170°C. Canonical correlation analysis (CCorA) was performed using *XLSTAT2014* software and a minimum filter factor of 80%. Variables were standardised (mean-centred and then divided by the sample standard deviation) prior to conducting this analysis. Y1 (red) sunflower oil samples; Y2 (green) corresponding fried potato chip samples.

The CCorA model applied revealed that there was only a single canonical dimension between the two datasets, which was found to be very highly significant (*p* <10^−6^, Wilks'-Lambda test); this first dimension corresponded to a canonical correlation of 0.99, which accounted for 67.2% of the total variance. Figure 2 shows a canonical correlation plot of the second vs. the first factors arising from this analysis, which shows that this first CCorA dimension (F1) featured strong positive correlations between the heated sunflower oil source *n*-alkanal and *trans,trans*-alka-2,4-dienal concentrations, and the increasing frying episode reuse code values, with the levels of these unsaturated aldehydes present in potato chip samples fried for durations of 0–80 min. However, potato chip *trans*-2-alkenal contents were less strongly correlated with these variables, and it appeared that oil *n*-alkanal concentrations were marginally anticorrelated with those of potato chips. This latter observation is presumably a reflection of the higher proportionate levels of saturated aldehydes present in the potato chip samples, as previously noted by our group (47), and others (66).

## Comparative Evaluations of the Biochemical Reactivities and Metabolism of Aldehydes

Although specific rates and thermodynamic favourabilities of the reactions of non-acrolein α,β-unsaturated aldehydes with biomolecules will differ somewhat from those of acrolein itself, they remain extremely chemically-reactive, most notably their abilities to form adducts with critical thiol functions in proteins and other biomolecules via Michael addition reactions, along with their abilities to alkylate DNA base moieties. Despite differences in these relative chemical reactivities, the mutagenicities of these toxins are all similar, i.e., log.TA100 mutagenic potency (logarithm of revertants/μmol). In Salmonella, TA100 values are: acrolein (3.38); crotonaldehyde (3.29); *trans*-2-pentenal, -hexenal and -heptenal (3.14, 2.99 and 2.67, respectively); and hexa-2,4-dienal (3.27) (16). In any case, from this work it was concluded that genotoxicities/biological activities of this class of aldehydes were not influenced by their chemical reactivities; however, second-order rate constants for the reactions of acrolein, crotonaldehyde and *trans*-2-pentenal with glutathione (GSH) decrease somewhat in that order (67). Differences in their genotoxicities were found to be determined by their elctrophilicities, hydrophobicities, and bulkiness—overall, increasing hydrophobicity and bulkiness (steric hindrance) gives rise to lower mutagenic activities. Notwithstanding, all α,β-unsaturated aldehydes have sufficient levels of reactivity to be bioactive, especially with regard to the formation of adducts with critical proteins and DNA. Moreover, it is now fully appreciated that such aldehydes may diffuse away from their site of impact (from dietary sources), or generation (for *in vivo*-produced sources), to react with more remote biomolecular substrates (67), and hence act as secondary toxic messengers in this context; therefore, it may be argued that the lower their chemical reactivities, the more remote this diffusion process, and the more likely their ability to react with and chemically modify a broader spectrum of critical biomolecules.

The toxicological actions and effects of α,β-unsaturated aldehydes are markedly complicated by the wide range of metabolic pathway routes for their transformations available to these agents *in vivo*, and these are illustrated for acrolein here. This aldehyde, which has a high level of solubility in aqueous environments, may readily enter tissues where it forms Michael addition adducts with GSH, cysteine, N-acetylcysteine and thioredoxin; acrolein-GSH conjugates may be formed with or without a glutathione-S-transferase catalyst, and can subsequently be metabolised to cysteine- and N-acetylcysteine-conjugates through enzymatic cleavage of the GSH moiety's glutamate and glycine residues. In additional metabolic steps, aldehyde dehydrogenase oxidises the aldehyde/aldehyde hydrate function to a carboxylate anion derivative (2-carboxyethylmercapturate), or via the actions of aldoketose reductase, forms S-(3-hydroxypropyl)-N-acetylcysteine. However, alternative metabolic by-products of acrolein are acrylate and glycidaldehyde, but ***only*** if the oxidation and epoxidation processes involved, respectively, precede adduct formation with the above endogenous thiol compounds. Similar complex metabolic transformation routes exist for higher homologues of acrolein such as *trans*-2-nonenal, which, like other α,β-unsaturated aldehydes, are absorbed from the gut into the systemic circulation and excreted in the urine as mercapturate metabolites *in vivo* (12).

Metabolic routes for the transformation of structurally simpler *n*-alkanals such as *n*-hexanal to less toxic and readily excretable carboxylic acid anion or alcohol products are available in the human body, and these include aldehyde oxidases, aldoketoreductases, alcohol dehydrogenases, aldehyde dehydrogenases/reductases and aldehyde dehydrogenases (ALDHs), along with cytochrome P450 enzymes (68). Such systems appear to be very effective at diminishing aldehyde concentrations *in vivo*. Notably, a total of 20 operational ALDH genes located at remote chromosomal localisations express 20 ALDH protein species, and these comprise the human ALDH superfamily; these proteins are members of 11 differing ALDH families. A full, up-to-date review of the *in vivo* absorption and metabolism of aldehydes in general, including MDA, may be found in Ahmed Laskar and Younus (52).

If acrolein or other α,β-unsaturated aldehydes were chemically-reactive at their site of *in vivo* administration, which may indeed be the case, these processes would be critically dependent on the availability of “aldehyde scavengers” such as GSH at the sites involved. In any case, it is exactly this high level of chemical reactivity which renders these secondary LOPs toxic and mutagenic, most notably toward critical biomolecules such as DNA.

## Toxicities of α,β-Unsaturated Aldehyde Homologues

### Toxicological Overview

Aldehydes are ubiquitous environmental toxins, and humans are continuously exposed to a plethora of aldehyde admixtures, their compositions being highly dependent on their particular sources, e.g. as food additives, fried and other foods, frying oil fumes, cigarette smoke, alcoholic beverages etc., which are also determined by factors such as the occupation, personal and religious habits, and geographical residence of those exposed. However, despite this, governmental legislation and research investigations tend to be targeted on the toxicity of individual aldehydes only, and not complex environmentally-available admixtures, and hence these only provide a limited scope of their human toxicological potential. Each aldehyde toxin therefore could contribute toward collective adduct formation, which could give rise to an additive pattern of toxicity. Therefore, exposure to such environmental aldehyde admixtures, particularly those containing the more toxic α,β-unsaturated classifications, and especially at the high human-uptaken levels noted above in Table 2, could constitute a highly significant overall public health risk potential. The total α,β-unsaturated aldehyde contents of French fries (predominantly but not exclusively *trans*-2-alkenals, and *trans,trans*- and *cis,trans*-isomers of alka-2.4-dienals) is approximately double that of *trans*-2-alkenals alone, for which acrolein can be considered the lowest, albeit non-isomeric, homologue. Hence, estimates of the contents of aldehydes in the proposed maximal 60 g potato chip/day allocation individually will only represent about one-half of this total unsaturated aldehyde pool of reactivity and toxicity.

Furthermore, since α,β-unsaturated aldehydes represent a sub-class of conjugated type-2 alkene compounds, they may, in principle, exert type-2-alkene toxicological actions, i.e., such alkenes share a common mechanism for this process. Indeed, toxicological actions exerted by human exposure to complex admixtures of dietary-derived α,β-unsaturated aldehydes and alternative type-2 alkenes, both prior to and following their *in vivo* absorption into the systemic circulation, is presumably ascribable to their combined individual rates and extents of protein thiol adduct generation, which would be expected to be dependent on composites of their individual electrophobicities and steric hindrances, for example.

Acceptable daily intake (ADI) (equivalent to Tolerable daily intake, abbreviated TDI) and no adverse effect level (NOAEL) values for acrolein, metaldehyde and acrylamide, and the potently toxic herbicide paraquat, are provided in Table 4 below [data extracted from (69)]. For acrolein, a NOAEL value of 0.05 mg/kg BW/day was derived from a 2-year gavage rat investigation, with mortality and serum biochemical effects observed at the next highest dose level applied. Of the 504 tabular entries available for such toxins in the complete document (69), only 31 had lower ADI values than acrolein, and only 13 had equivalent values to it. Importantly, the ADI and NOAEL values of the herbicide paraquat are both nearly 10-fold greater than those of acrolein.

**Table 4:** ADI and NOAEL values of acrolein, metaldehyde, acrylamide, and the weed-killer paraquat (mg/kg BW per day) (69).

**Toxin**	**ADI (mg/kg BW/day)**	**NOAEL (mg/kg BW/day)**	**Date**
Acrolein	0.0005	0.05	15/03/94
Metaldehyde	0.005	5	11/09/86
Acrylamide	0.04	0.50^*^	25–27/06/03
Paraquat	0.004 (as its cation)	0.45	27/06/03

*The registered date of these observations is also provided; those made for acrylamide were obtained from Acceptable Daily Intakes (70)*.

* *Value for peripheral neuropathy*.

The margin of exposure (MOE) values (equation 1) of aldehydes, and LOPs in general, should be employed in risk determinations, since these consider the benchmark dose lower 95% confidence limit (BMDL_10_), a parameter which represents this specified value of the amount (dose) of a test substance which gives rise to the occurrence of a toxic effect when expressed relative to that of a negative control (EDI represents estimated daily intake). MOE values which are lower than 10,000 indicate a potential WHO-defined health risk (71).

MOE = BMDL_10_ (μg/kg BW/day)/EDI (μg/kg BW/day)

From ATSDR (72), the BMDL_10_ value for acrolein is 360 μg/kg BW/day. On the basis of this figure, an estimated MOE value for acrolein is only 360/34 = 10.6, using the above 34 μg/kg BW EDI estimate in Lawson (34). In fact, if we use the suggested 70 μg/day total human acrolein intake estimate (1.0 μg/kg/day for a 70 kg BW subject) instead, this MOE value would still be substantially lower than the specified WHO 10,000 limit (i.e., 360). Therefore, in the author's view, acrolein exposure represents a major cause for public health concern. Moreover, particularly astonishing is the knowledge that acrolein is only one out of a myriad of secondary (and tertiary) aldehydic LOPs, including the α,β-unsaturated class alone (Table 2), and as noted above, human consumption of variable toxic levels of quite a wide range of dietary aldehydes in fried foods simultaneously, or inhalation of them in cooking oil fumes, will markedly exacerbate such toxic effects (tertiary aldehydic LOPs are considered here to represent those generated from the thermal deterioration of secondary ones, for example the degradation of longer chain alka-2,4-dienals to shorter *n*-alkanals such as acetaldehyde, acrolein and *trans*-2-alkenals). Indeed, the concurrent assault of many aldehydes, which are of similar chemical reactivities and toxicities, will be expected to substantiate and perhaps even polarise the variety of adverse health effects associated with their excessive exposure, e.g., those documented in Grootveld et al. (12), Penumetcha et al. (13), Staprãns et al. (14), Stavridis (15), Benigni et al. (16), Long et al. (17), Grootveld et al. (18), Benedetti et al. (19), Indart et al. (20), Jayaraj et al. (21), Yang et al. (22), Zheng et al. (23), and Brühl et al. (24).

In 2009, Lachenmeier et al. (73) found that the corresponding MOE value for acetaldehyde from alcoholic beverage sources alone was 498, an observation again presenting a considerable public health concern. Moreover, its life-time cancer risk was estimated to be 7.6 in 10,000, and this value markedly exceeded those of documented established ones from environmental contaminants, which generally lie between 10^−6^ and 10^−4^.

Unfortunately, many assessments of the toxicities and toxicologies of α,β-unsaturated aldehydes have focused on their very limited dietary availabilities as food flavouring agents only, and it appears that such important issues have only been dealt with in a perfunctory manner by the food industry. In 2003, the WHO reported that there was “No safety concern at current levels of intake when deca-(*trans,trans*)-2,4-dienal was used as a flavouring agent” (74), i.e., ***only when used as a flavouring agent***, and hence this conclusion was made without any quantitative consideration of its availability in fried foods or cooking oil fumes, nor other environmental sources of it (e.g., emission exhausts from restaurants). One further reference (75) is a document dated 2004, and again is focused only on the limited employment of α,β-unsaturated aldehydes as ***food additives only***. In this report, estimated daily intakes of 2-hexenal in Europe and the USA were 791 and 409 μg/person, respectively, values equivalent to only 11 and 6 μg/kg BW for a 70 kg human body mass.

However, version 52 of this reference (74) clearly states that ‘The *alpha,beta-*unsaturated dienals and related alcohols (Nos. 1173–1175, 1179–1181, 1183, 1185, 1189, 1190, 1195, 1196, and 1198) in this group ***cannot be predicted to be metabolised to innocuous*** ***products***. The evaluation of these 13 flavouring agents therefore proceeded via the B-side of the Procedure.' Such dienals include those commonly generated from the peroxidation of linoleoyl- and linolenoylglycerols (deca-(*trans,trans*)-2,4-dienal and hexa-(*trans,trans*)-2,4-dienal, respectively). Although dependent on frying oil PUFA contents, amongst other factors such as frying temperature and frying practise type, the contents of such alka-(*trans,trans*)-2,4-dienals in French fries may be as high as or higher than 60 molar % of their total aldehyde content (47).

Deca-(*trans,trans*)-2,4-dienal represents 50–60% of the total production of linear aliphatic alkadienal flavouring agents in both Europe and the USA (75), and estimates of its daily human intake as a flavourant are *ca*. 20 μg in Europe and *ca*. 70 μg in the USA. However, the potential role of this aldehyde as an indoor air pollutant toxin present in cooking oil fumes, particularly those derived from wok cooking episodes, has long been associated with the development of lung cancer in non-smoking restaurant employees (76–78).

More recently in 2017, the FGE.19 EFSA Panel on Food Contact Materials, Enzymes, Flavourings and Processing Aids concluded that “4,5-epoxydec-2-(*trans*)-enal [FL-no: 16.071] does raise a safety concern with respect to genotoxicity and therefore, it cannot be evaluated according to the Procedure” (79). As noted in our studies (47, 63), 4,5-epoxy-*trans*-2-alkenals represent ~8 molar % of the total α,β-unsaturated aldehyde contents of PUFA-rich corn or sunflower oils when thermally-stressed according to laboratory-simulated shallow frying episodes at 180°C. Therefore, the classification of α,β-unsaturated aldehydes in general as “Cramer class 1” substances is dubious, to say the least, since these epoxy-aldehydes, and possibly also alka-2,4-(*trans,trans*)-dienals, remain distinct and are not classifiable as such, especially with regard to the former's documented genotoxic actions. The suitability of the Cramer classification system with respect to secondary aldehydic LOPs is further discussed in section 5.4 below.

In 2015, the Texas Commission on Environmental Quality (TCEQ) completed a chronic non-cancer inhalation toxicity evaluation for crotonaldehyde, the next higher *trans*-2-alkenal homologue to acrolein, for the purpose of offering protection to the general public against any deleterious health effects arising from chronic exposure to this agent in ambient air (80). This study is, of course, of much relevance to the adverse health risks and effects presented by the inhalation of aldehydic LOPs present in cooking oil fumes during domestic frying or wok cooking practises. In view of the limited availability of toxicity data for this aldehyde, acrolein was employed as an indexing agent to primarily establish a crotonaldehyde:acrolein relative potency factor (RPF), from which a reference value (ReV) was determined. Investigations comparatively featuring the adverse toxicological effects of both crotonaldehyde and acrolein only were employed to compute the above RPF. From the *in vivo* investigations reviewed, which monitored a 50% respiratory depression in rats and two mice species, together with a 50% subcutaneous lethality in both rats and mice, an RPF value of 3.0 was calculated. Similarly, an evaluation of available *in vitro* cell culture data, which explored the cytotoxic actions of these aldehydes toward normal human lung fibroblast and mouse lymphocyte cultures, also gave rise to an *in vitro* RPF of 3, which agreed with the above *in vivo* value. The TCEQ-derived chronic ReV for acrolein of only 1.2 ppb was therefore multiplied by the RPF of 3, generating a ReV for crotonaldehyde of 3.6 ppb (equivalent to only 10 μg/m^3^).

One study featured in this investigation and conducted in 1987 involved the administration of increasing doses of crotonaldehyde (2–40 mg/kg BW per day for 5 days per week), to groups of *n* = 10 F344 rats and *n* = 10 B6C3F1 mice (both male and female) via oral gavage in a corn oil vehicle for a total 13-week period (81). Overall, rats were found to be more susceptible to the adverse effects exerted by this aldehyde than were mice. At doses of ≥5 mg/kg/day, crotonaldehyde-related mortality was noted in both rat genders, and acute nasal cavity inflammation was observed in females. At a dose of 10 mg/kg/day, forestomach epithelial hyperplasia (microscopic lesions) were found in the stomach of rats. Crotonaldehyde-induced gross necroscopy lesions (i.e., thickened forestomach or nodules) were observed in both male and female rats, along with acute nasal cavity inflammation in males. Furthermore, at the 40 mg/kg/day dose, significantly diminished body weights were noted for male rats at termination; additionally, moderate necrosis, acute inflammation, forestomach hyperkeratosis and ulcer development were observed. Like acrolein, crotonaldehyde inaugurates major point-of-entry effects in view of its high chemical reactivity.

Perhaps surprisingly, for mice, no crotonaldehyde-mediated gross necroscopy lesions were found, although hyperplasia of the stomach epithelial lining (microscopic lesions) was noted.

### Feeding of Oxidised Frying Oils (Mostly PUFA-Rich Oils, Such as Corn, and Soybean Oils) to Production Animals (Pigs and Chickens)

PUFA deterioration during the lipid peroxidation process in production animal feeds gives rise to significant reductions in energy value (82), together with a series of adverse effects on animal health, their overall metabolic oxidative status, and retardations to their growth performance when receiving these lipid-rich diets, notably without the application of any measures implemented for controlling the induction of autocatalytic peroxidation processes (83). Indeed, the economic benefits proffered by the use of peroxidized and pre-used cooking oils as lipid and energy sources in animal feeds are, of course, negated by major concerns that the consumption of toxic aldehydes and other LOPs therein may negatively impact upon animal health, growth, and performance.

Production animals, e.g., swine and poultry, receive feed fats, and oils as a significant proportion of their dietary energy requirements, although it is important to note that for these products there remains a marked variation in their quality, overall composition and feeding value. Although lipid quality is generally assessed using moisture, unsaponifiables, insoluble materials, acidity titres and FFA contents, little or no information is required or provided regarding their LOP contents and overall feeding values. Indeed, it is well-known that the peroxidation of lipids in feed serves as a major quality index related to breeding animal growth performance and health. Unfortunately, the myriad of tests commonly employed for monitoring LOP generation in these food products appear to be limited to older or non-specific, uninformative methods such as the TBARS assay, and to date no maximum tolerable limits for these quality markers have been established, nor implemented for different lipid sources. To date, analysts are largely focussing on the combined use of peroxide and anisidine values, along with the TBARS test, in order to provide an acceptable, cost-effective indication of peroxidation in lipid feed products, although it is appreciated that almost no single method can satisfactorily ascertain and regulate its nature and extent. Notwithstanding, high-resolution ^1^H NMR analysis may serve to solve these problems, most notably since it can simultaneously monitor a wide range of primary, secondary and tertiary LOPs, but this specialist and somewhat restrictive and very bulky technique will clearly not be directly applicable at local test sites. However, the advent of non-stationary, compact low-field (LF) benchtop NMR facilities may render this analytical technology as a site-applicable tool for such assessments (84).

Notwithstanding, the acyl chain FA compositions of lipidic food matrices being assessed should also be considered, notably those focussed on estimations of the resistance or susceptibility of acylglycerol FAs to peroxidation. Whereas, methods such as the active oxygen, oxygen bomb, oil stability index and iodine value (IV) methods are quite valuable for such tasks, both multianalyte LF and high-field (HF) NMR analyses have the ability to determine the individual FA compositions of such feeds, in addition to any LOPs detectable, and a combination of both sets of analytes allow thorough and reliable assessments of the extent of peroxidation and hence risk status of the products explored. Moreover, the greater sensitivity of the HF NMR technology permits the distinction of differential patterns of aldehydic LOPs arising from different types of frying oils.

In 2016, Shurson et al. (85) systematically reviewed 16 published studies on pigs, and found that mean reductions of 11.4% in growth rate, and 8.8% of feed intake in these animals when fed diets containing peroxidized lipids than isocaloric ones comprising unperoxidised forms of the same types of lipids. Additionally, blood serum α-TOH levels were decreased, and serum TBARS content was enhanced with peroxidized lipid feedings. Although the TBARS method for monitoring peroxidation in its current form is considered completely inappropriate and valueless by this author, these studies were interpreted to indicate that the feeding of peroxidized lipids deleteriously affects the metabolic oxidative status of pigs. Although these researchers maintained that it would be unclear if lipid-soluble chain-breaking antioxidants could be useful additions to lipidic energy diets in order to achieve their capital nutritional value, or indeed if their addition to swine dietary sources is valuable for combating oxidative challenges posed by the consumption of peroxidised lipid-containing foods.

Since energy represents the most costly component of swine diets, nutritionists are now attempting to optimise the calorific value of ingredients in commercial production animal feeds, in view of the major requirement of nutritionists for accurate, reliable, reproducible and validated analytical methods and techniques for the quantification of selected LOPs in feed products prior to investigating the influence of dietary lipid peroxidation on the growth and oxidative metabolism characteristics of production animals. Clearly, the above NMR analysis strategies may offer much potential for evaluating lipid integrity, quality and peroxidation in such samples.

A survey of lipid quality in the Midwest U.S.A. region reported by Shurson et al. (85) revealed that lipid materials derived from a local feed mill had total moisture, insoluble materials and unsaponifiables ranging from 0.8 to 3.7% (w/w), active oxygen method values ranging from 8.0 to 332 h, IV values from 66.3 to 84.0 g/100 g lipid, peroxide values (PV) from 0.4 to 7.3 mEq/kg, and FFA levels from 5.8 to as much as 51.6% (w/w). Therefore, these results confirm wide variabilities in the quality and compositional status of lipids which were being fed to poultry and livestock. Additionally, information regarding the relative merits of each lipid quality measure on both digestible and metabolizable energy (DE and ME, respectively) contents, and the nutrient use of lipids, remains sparse.

Unfortunately, lipids and blended lipid agents obtainable in the animal feed ingredient market, markedly vary in their FA and energy contents, and overall quality, along with price. Therefore, the above lipid peroxidation and other lipid quality monitoring assays are employed to certify that such products satisfy trading specifications; however, little or no information is actually provided on the nature and extent of lipid peroxidation therein, nor on their respective feeding values.

Lipid sources that contain high concentrations of polyunsaturated fatty acids (PUFA) are highly susceptible to peroxidation, especially when exposed to heat, light, oxygen, and transition metals during production, processing, and storage (86).

Unfortunately, for such investigations there remains no single assay system with the ability to fully characterise the nature and extent of peroxidation from all possible lipid sources, although suggestions for the applications of LF or HF spectrometers for this purpose made here should serve to provide effective solutions these problems. In view of these issues, to date the prediction of negative health effects and growth performance characteristics in animals fed diets containing LOPS below or above acceptable limits, remains problematical. Indeed, currently there is a major requirement for such limits to be established. Whereas, a number of investigators have suggested minimal threshold levels of dietary peroxidation that significantly diminishes growth performance in animals (87–90), no generally acceptable standards have yet been developed.

Recently, Yuan et al. (91) explored food quality markers for the determination of animal health and performance in non-ruminant animals in a model featuring the feeding of an oxidised oil product to broilers and nursery pigs. Indeed, for the first time, these researchers examined potential correlations between the chemical profiles of oxidised oils in feed sources and the growth performance of these animals. For this purpose, six thermally-oxidised soybean oil products, exposed to processing periods and temperatures ranging from 336 hr. at 45°C, to 10.5 hr. at 225°C, were prepared, and both animal species were randomly allocated to groups consuming diets which contained these. The peroxidation status of these oils was determined by standard indicative assays such as peroxide, *p*-anisidine, TBARS and oxidised FA values, and also by LC-MS analysis coupled with chemometric analysis. Animal performance was evaluated via the monitoring of body weight gain, feed intake, and gain-to-feed ratio indices. Results acquired demonstrated that oil p-anisidine values exerted the greatest influence on the growth performance of both non-ruminant animals; free and oxidised FAs were rated second and third in terms of their inverse correlations with the above performance and well-being indices. Of the 17 aldehydes determined in oxidised sunflower oils using LC-MS analysis, the C9-C11 alkenals, most notably 2-decenal and 2- undecenal, had the highest anti-correlations with animal growth performance (*r* <-0.80) than those observed for C5-C8 saturated alkanals. These data indicated that the toxicity of aldehydic LOPs was dependent on both their unsaturation status and homologue chain length. Since these C9-C11 α,β-unsaturated aldehydes represent major aldehydic LOPs contributing to *p*-anisidine values, these toxins may serve as valuable predictive growth and performance markers, and also as indicators for the intake of peroxidised oil-containing feeds, in non-ruminants. However, in view of its relatively higher omega−3 FA content, when peroxidised, soybean oil has been found to generate aldehydes derived from the fragmentation of this class of FA, and these include low-molecular-mass *n*-alkanals and 4-oxo-*n*-alkanals, along with malondialdehyde and acrolein (48).

In view of these observations, the author proposes that the determination of a broad spectrum of aldehydic LOPs as biomarkers, and also their metabolites, in biofluids such as blood plasma and urine collected from such animals involved in feeding regimens containing used frying oil supplements, would serve to provide much valuable information regarding their health, performance and longevity.

In a study involving poultry feed, Selim and Hussein (92) explored the effect of incorporating sugar beet pulp (SBP) in the diets of *n* = 200 laying hens on their performance, the quality of and lipid peroxidation in eggs layed, and blood clinical chemistry parameters. Four groups with dietary supplementations of 0, 3, 5, and 7% (w/w) SBP were compared. Results acquired provided evidence that the dietary inclusion of SBP linearly (*p* < 0.01) enhanced feed intake, egg generation, egg weight and mass, and also was effective in ameliorating feed conversion ratio, and the yolk colour core and Haugh unit. Eggs laid by hens fed SBP laid eggs which had enhanced protein content and diminished ether extract values. Dietary SBP was also found to reduce egg yolk MDA, cholesterol and TAG contents, observations which were accompanied by elevated glutathione peroxidase activity. Moreover, hens receiving dietary SBP had significantly decreased serum total lipids, cholesterol, alanine aminotransferase, aspartate aminotransferase, and creatinine levels. Therefore, the authors concluded that the dietary incorporation of SBP may indeed enhance hen health and performance, along with egg quality and its shelf-life. However, further experiments are required to establish whether any chain-breaking antioxidants present in SBP are actually improving the quality of the poultry feed employed, and preventing lipid peroxidation therein *ex-vivo* prior to hen feeding, or indeed whether such SBP antioxidants or other nutrients exert their effects *in vivo*.

### Model Experimental Investigations Relating the Intake of Fried Foods and/or Aldehydic LOPs to Human Disease Pathogenesis

As early as 1999, Woutersen et al. (93) reviewed both animal model and epidemiological reported investigations focused on the effects of dietary fat consumption on the risks of breast, colorectal, pancreatic, and prostate cancers. These studies revealed that its intake exerted effects on prostaglandin and leukotriene biosynthetic pathways, and that these served as universal mechanisms for these deleterious health effects.

Some animal studies have explored the genotoxic and oxidative stress potential of mutagens detectable in thermally-stressed soyabean and sunflower oils, and lard (94), and these studies revealed increases in liver and blood serum peroxidation markers, together with modifications in lipoprotein compositions in rats by the consumption of thermo-oxidised sunflower oil (95). Moreover, the short-term feeding of unheated culinary oils containing strain T100 mutagen and linoleate peroxidation products to rats gave rise to observations of cellular damage to kidneys and liver, and also promoted urinary mutagenicity and oseophageal cell proliferation (96).

In 1991, Feron et al. (52) evaluated the carcinogenic potential, mechanism of action, and risk assessment of dietary aldehydes, and they found that these effects for acetaldehyde, crotonaldehyde and furfural were all potentially significant. However, they also found that acrolein, citral, formaldehyde and vanillin offered no serious dietary risk factors. These studies also revealed that acetaldehyde, crotonaldehyde and furfural should be assessed for their cytogenic, cytotoxic and mutagenic properties, with special considerations of their human exposure levels and mechanisms of their toxic actions.

Formate also serves as terminal LOP, and as formic acid, it can be generated from the decomposition of MDA in oil media, along with acetaldehyde and an MDA dimer (97). Adverse health effects manifested by inhaled formaldehyde on the prominence of oxidants and antioxidants in rat cerebellum throughout the postnatal development process have been previously explored (98), and this investigation revealed that MDA and nitric oxide (NO) concentrations, together with glutathione peroxidase activity, were upregulated, although t-superoxide dismutase (SOD) activities were notably reduced in rats exposed to formaldehyde. Indeed, these effects may indicate irreversible oxidative stress and toxicity induced by inhalation of this simple aldehyde.

Evidence available indicates that the *in vivo* accumulation of reactive aldehydic LOPs appears to represent neurodegenerative mediators in both Alzheimer's and Parkinson's diseases, amyotrophic lateral sclerosis and multiple sclerosis (99, 100). Moreover, the involvement of such aldehyde build-ups in the periphery of the CNS have been confirmed for all these neurological conditions. Special relationships between acetaldehyde and Parkinsonism, with particular relevance to the activities of cytochrome P450 and its CYP 2E1 isoenzyme systems, were investigated by Vaglini et al. (101). Acetaldehyde augments the development of Parkinsonism in mice via the actions of the 1-methyl-4-phenyl-1,2,3,6-tetrahydropyridine neurotoxin. Additionally, postulates on the pathogenesis of autism spectrum disorder (ASD) are remarkably consistent with aldehyde toxicity, and these involve the accumulation of both endogenous and exogenous classes of these toxins from the mutation of critical aldehyde-neutralising enzymes, as observed in alternative neurodegenerative diseases (102). This hypothesis was powerfully bolstered by a highly intensive critical review of available biochemical, genomic and nutritional data, and to date has had a strong impact on the diagnosis, treatment and potential prevention of ASD.

Interestingly, guinea pigs exposed to acrolein and formaldehyde at concentrations relevant to those in homes and workplaces, along with excessive doses (0.3 and 0.1–31 ppm, respectively) for durations of 2 or 8 h, revealed that a formaldehyde level of 9 ppm was necessary for the induction of airway constriction; this concentration is equivalent to that generated by acrolein within a 2 hr. exposure to 0.30 ppm of this aldehyde (103).

Nevertheless, an 8 h exposure period to only 1 ppm formaldehyde induced an immediate airways constriction similar to that resulting from a 2 h. one to higher levels of this toxin (9 or 30 ppm).

Notably, such aldehyde inhalation induces long-lasting airways effects, and therefore these observations have an important and wide-ranging broad public health effect, most especially for individuals with sensitive airways, for example those with asthma. Deleterious health effects associated with the dietary intake of a repeatedly-reused, pre-refined vegetable frying oil on blood biochemistry, antioxidant enzyme concentrations, and histopathological indices of Wistar rats was investigated by Ventaka and Subramanyam (104). These experiments revealed that a 3-fold consumption of such oils exerted a cogent level of damage to jejenum, colon and liver of these animals, and also significantly modified selected antioxidant enzyme activities. Furthermore, upregulated blood concentrations of glucose, creatinine and cholesterol were observed, as were downregulated total protein and albumin levels.

Additionally, a number of reports have revealed elevated arterial blood pressure indices, together with other deleterious health effects in animal model system experiments. In 1996 and 2008, Osim et al. (105), and Leong et al. (106), respectively, discovered that administration of pre-heated palm oil to rats resulted in significant blood pressure increases, along with perturbances in cardiac muscle. Furthermore, Owu et al. (107) studied the effects of the chronic intake of diets containing 15% (w/w) fresh or thermally-oxidised palm oil on rat aorta in accordance with standard organ protocols, and found that administration of the latter gave rise to functional alterations therein. Interestingly, they also revealed that significantly increased mean arterial pressure (MAP) arose from consumption of the diet containing the thermo-oxidatively stressed oil, but not in those receiving the unheated palm oil negative control; this observation was related to enhanced low-density-lipoprotein (LDL)-associated and total cholesterol levels, which predisposes to hypertension. A further investigation (108) revealed that a thermo-oxidised palm oil diet appeared to damage liver cells more so than that noted with feeding of the unheated control product.

### Tolerable and Acceptable Human Daily Intake Limits Set for Selected Aldehydes by Governmental Health Authorities and the WHO

The WHO's tolerable daily intake (TDI) value for acrolein (109), which represents the simplest α,β-unsaturated aldehyde available, is 525 μg/day (9.36 μmol./day) for a 70 kg average BW human, which differs quite substantially from the Australian Government Department of Health's (AGDH's) acceptable daily intake (ADI) one, which is only 35 μg/day (0.62 μmol./day) (110). To remain impartial, both limits were reported by us in Moumtaz et al. (47). Notwithstanding, it must remain very difficult for scientists, let alone the public in general, to understand global differences and possible disagreements between these established, reputably-sourced figures. Which one is more acceptable? As noted above, the ADI value is exactly equivalent to the TDI one, but it appears that the former is employed for food additives (111), and the latter for food contaminants, but not exclusively so. Notwithstanding, the US Environmental Protection Agency has now replaced both the ADI and TDI parameters with a single index termed reference dose (RfD).

Therefore, from molar concentration values available in Moumtaz et al. (47), calculated mean *n*-alkanal, *trans*-2-alkenal and *trans,trans*-alka-2,4-dienal contents for the UK's 30 g average daily French fry consumption level are *ca*. 4, 5 and 4 μmoles/day, respectively. Hence, although total estimated intakes for the more toxic α,β-unsaturated classes of aldehyde (9 μmol./day) are very similar to the WHO's TDI index for acrolein, this composite *trans*-2-alkenal and *trans,trans*-alka-2,4-dienal content value is nearly 15-fold higher than the corresponding AGDH one for acrolein in the context of both reactive aldehyde-CHO and electrophilic -CH=CH-CHO C-3 function equivalents. Similarly, a consideration of this 30 g average daily consumption quantity for global fast-food chain French fry servings yields a total human α,β-unsaturated aldehyde consumption rate as high as 17 μmoles/day from the investigation reported in Le Gresley et al. (63), a value which is *ca*. 1.8- and a striking nearly 30-fold higher than the above WHO and AGDH daily consumption limits, respectively. Intriguingly, this total value includes lower levels of the even more reactive and toxic substituted α,β-unsaturated aldehydes 4,5-epoxy-*trans*-2-alkenals, 4-hydroxy- and 4-hydroperoxy-*trans*-2-alkenals (together ~7% of the total level found in French fry products); these aldehydes were detectable in French fry samples analysed and not in those of the investigation described in Moumtaz et al. (47) in view of the higher sensitivity and selectivity of the 600 MHz NMR spectrometer facility employed for these analyses in the former investigation. Furthermore, these estimates only feature α,β-unsaturated aldehydes, and not the less reactive and plausibly less toxic saturated aldehydes comprising *ca*. 25% of the total available.

It is also of much importance to note that these estimated intake values would be exactly double those computed above if we employed the suggested “worst-case scenario” French fry consumption rate of 60 g/day in the UK in place of the average 30 g/day figure. Moreover, along with French fries, if we also followed the human consumption of a range of alternative fried food products, such as those reported in Spain (55) with a mean total fried food consumption rate of 138 g/day, this worst-case scenario level of 60 g/day for French fries alone would this time be more than doubled. Additionally, we haven't even considered the inhalation of aldehyde-enriched cooking oil fumes from frying episodes yet, especially those arising from shallow frying and wok cooking practises.

Assuming that the *n*-alkanal, *trans*-2-alkenal and *trans,trans*-alka-2,4-dienals thermally-generated in frying oils are exclusively the only peroxidation products arising from linoleoylglycerols, i.e., *n*-hexanal, *trans*-2-octenal and deca-(*trans,trans*)-2,4-dienal, respectively, then data from Velasco et al. (26) yields estimated 370, 460, and 730 μg quantities, respectively, per average 30 g daily French fry consumption amount in the UK; 76% by weight of these are the more toxic α,β-unsaturated classes (“worst case scenario” 60 g/day values would obviously be exactly 2-fold higher). For a typical ‘large’ 154 g portion, these values would be 1.91 (1.08) mg *n*-hexanal, 2.37 (1.040) mg *trans*-2-octenal, and 3.76 (1.40) mg *trans,trans*-alka-2,4-dienal, respectively, (acrolein mass-equivalent values are provided in brackets, but clearly molar equivalents are more appropriate units to employ since this directly relates to the “molecular mass-normalised” quantities of reactive and toxicologically significant aldehyde-CHO and thiolate/amine function nucleophile-susceptible -CH=CH-CHO C-3 groups available).

*In vivo* concentrations of xenobiotics are, of course, determined by the amounts of oral doses administered in toxicological screening experiments. Notwithstanding, the 7.5 μg/kg/day TDI value specified for acrolein by the WHO (69, 109) is <4-fold lower than the 34 μg/kg/day estimate for the daily dietary acrolein intake provided in (62). It is understood that the figure of 7.5 μg/kg/day was deduced from a tolerable concentration of 1.5 μg/mL, which in turn was computed after applying a 100-fold “safety factor” to a NOAEL value for non-neoplastic lesions in the GI tract of rodents of a 150 μg/mL solution administered via the gavage system (and/or sometimes in drinking water) (69): However, such lesions are dependent on solution concentration and not dose administered. As noted for other aldehydes administrated via the gavage route, acrolein is extremely irritating. Moreover, when tissues, for example the forestomach gastric mucosa, are repeatedly exposed to it, it exerts toxic actions (112). Additionally, the 90-day period involved for such testing is certainly not an entire lifespan for rodents, and nor is it for other species of laboratory animal involved in these investigations. The suitability of the gavage tube administration route for evaluating the toxicities of aldehydes in animal model experiments is discussed below.

### Relevance of Delivery Route for Aldehydes in Animal Model Toxicological Studies

Firstly, it should be noted that the oral gavage route of administration of aldehydes in rodents is not strictly relevant to their dietary intake in foods in humans. Indeed, such gavage exposures circumvent interactions of the oral mucosa with such human dietary toxins, and this gives rise to major differences in their absorption, metabolism and overall bio-availabilities. Further complications arise from the ability of the gavage system to perforate the oesophagus, and the ordination of stress responses, aspiration, and pulmonary injury (113). Taken together, these adverse phenomena may markedly affect toxicokinetic models, and hence overall confound the determination of ADI (TDI) parameters. Hence, it appears that such gavage delivery systems do not effectively model human dietary exposures to potential toxins.

Secondly, toxicological ADI/TDI values are typically expressed in **microgram** or **milligram masses** of the assessed agent/toxin per kg BW per day [i.e., μg/kg BW/day or mg/kg BW/day, respectively (111)]. However, the medium used for gavage delivery of potential toxins in question should also be considered as a potentially confounding factor for these evaluations. Indeed, are aldehydes administered as aqueous solutions via the oral gavage route relevant or not to those available in food sources, particularly those predominantly available in the oil/lipidic phases of fried foods such as French fries etc., i.e., that comprising frying process-uptaken, LOP-enriched culinary oils, say 10–15% by weight? Nevertheless, perhaps the gavage administration of aldehydes in culinary oil media, e.g., corn oil for crotonaldehyde as in Grant and Jenkins (81), will be less questionable and more relevant?

Thirdly, another key point is how exactly does the NOAEL concentration value for non-neoplastic lesions observed for acrolein in the rat [0.15 mg/mL (109)] relate to those of aldehydes in such fried food media? The amount delivered physiologically by the oral gavage route would obviously depend on acrolein solution concentration, the total volume delivered, and the rate and precise site of its delivery, amongst other factors for consideration.

Moreover, finally, the adverse health and irritant actions of acrolein and further α,β-unsaturated aldehydes are likely to be significantly influenced or perturbed by their reactivities with aldehyde-scavenging food molecules and/or physiological “masking” effects by them, in addition to the gavage administration route problems noted above. As noted, for ***specific*** analytical techniques applied, only the “free,” i.e., unadducted/unconsumed concentrations of such aldehydes, are determined in French fries by high-resolution ^1^H NMR analysis (47, 63), or for 4-hydroxy-*trans*-2-nonenal alone by high-performance liquid chromatography (HPLC) and HPLC/mass-spectrometric (MS) techniques (65), and therefore in principle they will have the ability to exert the same toxic or adverse effects as aqueous solutions containing the same level. In any case, Michael addition adducts of aldehydes with thiol function-containing biomolecules such as L-cysteine and GSH potentially serve as reserve sources of such toxic aldehydes (64, 114), which may enhance their longevity in living systems, as also noted in Moumtaz et al. (47). However, the *in vivo*, time-dependent passage of aldehydes from a predominantly lipid-based to aqueous medium is, of course, likely to exert some toxicodynamic/toxicokinetic effects; notwithstanding, living systems can be broadly chemically viewed as “mixed-solvent systems.” At the uptaken or administered levels, many aldehydes will be expected to have sufficient solubilities in aqueous media. In any case, it is this high level of reactivity of aldehydic LOPs with critical biomolecules such as proteins and DNA which is responsible for their toxicity and adverse health effects.

Therefore, the author accepts that there are expected to be some differences between the biological effects of concentrations applied via oral gavage administration and those consumed in fried or other food matrices, but any intake quantities above their specified ADI/TDI levels can be perceived to be those which potentially pose a risk to human health. This consideration is rendered relatively insignificant anyway in view of the now commonly-encountered underestimations of dietary aldehyde intake, as noted in European Union Risk Assessment Report (60).

In 1994, Hebert et al. (115) compared the administration of cinnamaldehyde (CNMA) to rats and mice via gavage tube in a corn oil vehicle (2-week dosing period) with that involving a microencapsulated form dosed from their feed (2 and 3 weeks for rats and mice, respectively). These food formulations contained daily doses equivalent to 0–3,000 and 0–10,000 mg CNMA/kg BW for rats and mice, respectively, and these were selected to provide doses which were approximately equivalent to that of the gavage-treated animals. Gavage dosings of ≥2,620 and ≥940 mg/kg/day and above in mice and rats, respectively, gave rise to virtually 100% mortality, but no deaths were observed in animals receiving the microencapsulated aldehyde. CNMA feed-dosed to rats and mice demonstrated a dose-dependent reduction in BW gain, which concurred with hypoplastic modifications in reproductive organs and accessory sex glands. Administration of CNMA by either of these administration systems gave rise to hyperplasia of the forestomach mucosa. The results acquired confirmed that the microencapsulation of potential toxins in animal feed represents a valuable alternative to the gavage delivery route investigations featuring the repeated dosing or prolonged exposure of agents tested. Indeed, this is justified by the observations that the toxic actions of CNMA were found to be similar to that found following gavage dosing, although the intake of feed-borne toxins is more relevant to human dietary exposures. The delivery of greater net doses of toxins was facilitated by this microencapsulation process, and in this context acutely toxic influences exerted by bolus doses are circumventable.

### Some Problems With the Cramer Classification System for Stratifying Aldehyde Toxicities

The Cramer classification system (116) has been the subject of much criticism and debate, with a sizeable proportion of the questions placed being ambiguous (117). However, this system clearly classifies “acrolein, methacrolein or their acetals” as Cramer structural class II compounds, and not less toxic class I agents. Moreover, “aliphatic compounds with 3 or more different types of functional groups” are classified as Cramer status III compounds, and this will presumably include the α,β-unsaturated aldehydic LOPs 4-hydroperoxy- and 4-hydroxy-*trans*-2-alkenals, and 4,5-epoxy-*trans*-2-alkenals, the rationale for this being that these are “complex molecules, the toxicity of which cannot be readily predicted” (116); these aldehydes are also generated from the peroxidation of PUFAs during high-temperature frying episodes, although generally at lower yields than the concentrations of *trans*-2-alkenals and alka-(*trans,trans*)-2,4-dienals formed (47). Additionally, epoxides, which undergo metabolic ring-opening and GSH conjugation are classified as Cramer class III, 3-membered heterocyclic ring compounds, and this classification must therefore also include toxic epoxy-FAs and their derivatives, which are also readily detectable at high levels in culinary frying oils and fried foods by ^1^H NMR analysis (47).

Additionally, for substances which “successfully” survive or by-pass the primary scrutiny applied to Cramer class I compounds, the Cramer class II classification confusingly includes agents which are “a common component of food or structurally closely related to a common component of food,” so that extremely broad requirement may indeed include selected classes of aldehydic toxins.

## Mutagenic, Genotoxic, and Carcinogenic Potential of Aldehydes

With regard to the mutagenic and genotoxic properties of aldehydic LOPs, it should be noted that there is now a long and expansive history of research work which has exactly focused on these arenas for more than 40 years or so, and notable progress made has been exhaustively reviewed by Eckl and Bresgen (118). So that readers are aware, the mutagenicity of chemical substances refers to their abilities and roles in engendering genetic mutations in base-pair DNA sequences, and for toxic aldehydes, this process usually involves their abilities to react with and hence covalently alkylate DNA base adducts. Conversely, genotoxicity arises from the abilities of such agents to damage intracellular genetic information, a process leading to mutations, which in turn may give rise to malignancies. However, teratogenesis, as observed with the feeding of post-thermally-stressed safflower frying oil to experimental animals (20), refers to the disturbed growth phenomena featured in the induction and generation of malformed neonates. Indeed, much valuable information is available in this area, with acrolein, malondialdehyde and 4-hydroxy-*trans*-2-nonenal being the most widely investigated, although there are some limitations with direct comparative evaluations of these results in view of the wide diversity of cell lines tested and their tissular sources. Notwithstanding, recent developments in the field of epigenetic effects, i.e., histon modification and DNA methylation, have provided results which are potentially highly significant, e.g. (119). Although not exerting any influence on the expression of both onco- and suppressor genes, *trans*-2-hexenal has displayed a cogent carcinogenic potential, which may be elucidated by its epigenetic actions (119); specifically, it appears to act as a non-genotoxic carcinogen. Notably, consistencies between the genotoxic and carcinogenic properties of agents are only about 90% (120). Therefore, the absence of genotoxic risks found for this reactive aldehyde (121) may be explicable by such epigenetic actions (120). The influence of the structural characteristics of α,β-unsaturated aldehydes on their mutagenicities and genotoxicities is explored in Esterbauer et al. (65), and results arising from this study are discussed in the section entitled Estimated Dietary Intakes of Acrolein and Other Aldehydes.

For acrolein, one report, which found no increases in tumour incidence in mice or rats when administered via the oral route, was made available in 1997. However, a version of the Environmental Protection Agency (EPA)'s Hazard Summary for Acrolein updated in September 2009 (122) stated that ‘***The potential carcinogenicity of acrolein cannot be*** ***determined because existing data are inadequate for an assessment of human carcinogenic*** ***potential for either the oral or inhalation routes of exposure’***, and referenced the US EPA's integrated risk information system for this purpose, so unless there are any more significant developments on this, it appears that the “official jury” is still undecided on the carcinogenicity of this unsaturated aldehyde when administered via the oral route.

However, in contrast, as early as 1986 Chung et al. (123) based at the Institute for Disease Prevention at the American Health Foundation orally-administered crotonaldehyde, which is mutagenic without metabolic activation, to F344 rats in drinking water at concentrations of either 0.60 or 6.00 mmol./L for a 113-week period, and histologically evaluated liver tumours in these groups against an untreated age-matched control one. At the lower dose level, crotonaldehyde was found to induce neoplastic lesions in the liver in 9/27 rats; a further 9 had neoplastic nodules, and 2 had hepatocellular carcinomas. At the higher dose level, however, this aldehyde gave rise to severe liver damage in 43% of these animals, with the remaining 57% developing abnormal liver cell foci. Although results acquired also indicated that crotonaldehyde was a weaker tumorigen than the established carcinogen N-nitrosopyrrolidine (NPYR), they provided strong evidence for its carcinogenicity. Indeed, the incidence of liver tumours in rats treated with crotonaldehyde and NPYR at equivalent doses (0.60 mmol./L) was 87 and 33%, respectively. For estimated dietary and wine drinking intakes of crotonaldehyde, a relatively high cancer risk of 0.1–1 cancer incidence/10^3^ humans, has been extrapolated from Chung et al.'s TD_50_ value (124). α,β-Unsaturated aldehyde concentrations of 0.60 and 6.00 mmol./L are actually quite low when compared to those of total *trans*-2-alkenals and more structurally complex unsaturated aldehydes found in vegetable-based culinary oils exposed to high-temperature frying practises, especially when repeatedly-used for this purpose (47) (a value of 13 mmol./kg was noted for sunflower oil exposed to thermal stressing at 180°C for only a 20 min period). Much further information focused on the established or potential carcinogenicity of aldehydes may be found in Eder and Budiawan (15).

In 1999, Eder et al. (125) conducted a risk assessment for crotonaldehyde and 2-hexenal (*cis*- or *trans*-isomers for these species were unspecified). Indeed, both these α,β-unsaturated aldehydes are mutagenic and genotoxic, and the former is also described as being carcinogenic, as noted above; indeed, both form 1,N2-propanodeoxyguanosine adducts on reaction with DNA. At that time, the highest daily intake of crotonaldehyde was considered to be that from cigarette smoke inhalation (31–169 μg/kg BW per day), whereas that from 2-hexenal was from dietary fruit and vegetables (31–165 μg/kg BW per day). In order to estimate cancer risk more reliably, these researchers developed and applied a sensitive ^32^P post-labelling strategy for determining these DNA adducts. In male Fischer rats dosed singly with 200 or 300 mg/kg BW crotonaldehyde orally via gavage tube (discussed below), such adducts were found in tissues, and in liver following repeated doses of 1 or 10 mg/kg BW. Cancer risk estimation using the method of Lutz (126), which evaluated correlations between the DNA adduct concentration-determined covalent binding index and the median toxic dose level (TD50) revealed a cancer risk of 1 in 10^7^ lives for 2-hexenal based on its above estimated daily intake. However, that for crotonaldehyde from Chung et al. (123), which arises from a cancer incidence of 0.07 at a daily dose level of 4.2 mg/kg BW derived from the smoking of 30 tobacco cigarettes per day, is equivalent to a risk of 0.58–1.8.0 new cases per 10^3^ new smokers, and this estimate is not dissimilar to that extrapolated in Chung et al. (124). Notwithstanding, the authors of Stavridis (125) surmised that the approach of Chung *et al*. may result in an overestimate of cancer risk arising from such crotonaldehyde exposure. Indeed, the binding studies performed in Stavridis (125) generated an estimate of this risk parameter which was 20-fold lower. However, such estimates did not also take into account the additive effects of dietary crotonaldehyde, plus a wide range of other α,β-unsaturated aldehydes, notably those arising from the ingestion of fried foods, commonly French fries.

Acetaldehyde is mutagenic ***and*** carcinogenic (127). Indeed, gene polymorphisms in aldehyde and alcohol dehydrogenase enzymes (ALDH2 and ADH, respectively), which are linked to an increased acetaldehyde exposure, substantially enhance cancer risk in alcohol drinkers, and these phenomena “**…*provide undisputable evidence*** ***for acetaldehyde being a local carcinogen not only in oesophageal but also in*** ***gastric cancer. Accordingly, acetaldehyde associated with alcoholic beverages*** ***has recently been classified as a Group 1 carcinogen to humans*.**” [quoted from Salaspuro (128)]. Indeed, this toxin gives rise to DNA damage, and it has further cancer-stimulating effects (129, 130). Furthermore, in dividing cells, acetaldehyde can be transformed to crotonaldehyde by polyamines, and it also generates mutagenic 1,N^2^-propanodeoxyguanosine derivatives (131). Since this less chemically reactive saturated aldehyde is a classified carcinogen, it should follow that more reactive α,β-unsaturated aldehydes should also have this classification; however, much further research work is required to fully establish this, with particular considerations of the environmental or food source quantities of these toxins ingested and absorbed *in vivo*. As specified above in Table 2, acetaldehyde may be classified as a tertiary LOP, since it can arise from the decomposition of alka-2,4-dienals.

Intriguingly, when applied dermally to mice and rats, the glycidaldehyde metabolite of acrolein exerts carcinogenic properties (132). Acrolein can also be transformed to malonate by liver aldehyde dehydrogenase. Moreover, many irreversible biotransformation processes which involve its reaction with cellular nucleophiles at the C-3 position to form stable conjugates appear to be responsible for acrolein's deleterious toxic effects. Overall, these metabolic routes are outlined in the section entitled Estimated dietary intakes of acrolein and other aldehydes, and are fully delineated in Moghe et al. (133).

Moreover, the report available in Wang et al. (16) clearly specifies the rodent carcinogenicities of crotonaldehyde, hexa-2,4-dienal, formaldehyde, acetaldehyde and glycidaldehyde, as do a range of further literature publications, for example (52, 79). In fact, in Stevens and Maier (134), the genotoxicity of unsaturated carbonyl compounds was demonstrated in eukaryotic cells, and some of these agents were indeed found to be carcinogenic.

The International Agency for Research on Cancer (IARC) has classified formaldehyde, another known aldehydic LOP, as a human carcinogen (135). Moreover, in 2011, the National Toxicology Program, an interagency program of the Department of Health and Human Services, also classified formaldehyde as a known human carcinogen in its *12*^*th*^ *Report on Carcinogens* (136).

Older studies have already demonstrated that crotonaldehyde is a liver carcinogen in rats, as noted above (15, 123), and malondialdehyde/(β-hydroxy acrolein) exerts cancer initiating activities in female Swiss mice (137, 138). Vinyl chloride, which is structurally related to acrolein, has been identified as a carcinogen in both animals and humans (139). Moreover, in cell culture experiments, acrolein has cytotoxic properties at concentrations of only 0.1 μmol./L (140), and the mutagenicity of such carbonyl compounds was fully established many years ago (141). Additionally, in 1992 Cohen et al. (142) demonstrated that acrolein had the ability to initiate urinary bladder carcinogenesis in rats.

More recently, Tan et al. (143) found that environmental and metabolic classes of aldehydes, notably formaldehyde and acetaldehyde, present health threats toward humans with heterozygous BRCA2 mutations, who have an enhanced susceptibility to cancer. This process occurs by the ready ability of aldehydes present at physiological concentrations to stimulate the degradation of BRCA2 protein, and hence predispose humans to cancer. In subjects with one faulty copy of the associated gene (~1 in 100 humans), this degradative process reduces this protein's activity below that required for efficacious DNA repair, a development facilitating cancer induction. Furthermore, Pontel et al. (144) have demonstrated that endogenous formaldehyde is a hematopoietic stem cell genotoxin and metabolic carcinogen.

Intriguingly, the genotoxic and carcinogenic risks associated with the dietary consumption of repeatedly-boiled sunflower oil by Wistar rats was explored by Srivastava et al. (145), and these researchers observed a dose-dependent ordination of aberrant cells and micronuclei, and significantly diminished antioxidant enzyme levels. This feeding exercise also modified hepatic foci, and gave rise to significant decreases in hepatic mass.

Of much significance, a very recent observation has provided a high level of evidence that aldehydes represent the dominant carcinogens present in tobacco smoke which give rise to DNA damage, inhibit DNA repair in tobacco smoke carcinogenesis, and also prevent many other tobacco smoke procarcinogens (including 4-(methylnitrosamine)1-(3-pyridyl)-1-butanone and polyaromatic hydrocarbons) from becoming DNA-damaging agents (146). On the basis of these results, the authors of this report proposed that toxic aldehydes represent the dominant tobacco smoke carcinogens. As documented in Moumtaz et al. (47), the aldehyde contents of a typical large size serving of restaurant French fries are not very dissimilar to those available for inhalation during the smoking of a 20–25 allocation of tobacco cigarettes (147).

In November 2020, a Working Group representing the International Agency for Research on Cancer (IARC) finalised their evaluations of the carcinogenic potentials of acrolein and crotonaldehyde, along with arecoline (the primary active ingredient of the areca nut, although not an aldehyde). Acrolein was classified as “probably carcinogenic to humans” (Group 2A) in view of “sufficient” evidence of carcinogenicity in experimental animals and “strong” mechanistic evidence. However, crotonaldehyde and arecoline were classified as “possibly carcinogenic to humans” (Group 2B) on the grounds of “strong” mechanistic evidence (148). Likewise, the US Environmental Protection Agency (EPA) has classified acrolein as a toxic pollutant with a significant priority, and their toxicological profile on this toxin is available in the IARC Monograph Working Group (149).

## Contemplations of Human Blood Plasma and Tissular Concentrations of Aldehydic LOPs: do They Have Endogenous or Exogenous Sources?

One major consideration is that, following their ingestion, *in vivo* absorption and metabolic transformation, will there remain sufficient toxic concentrations of chemically-reactive aldehydic LOPs such as acrolein in biofluids and tissues, and if so, will they then be able to penetrate into critical organ and cellular localisations where they have the capacity to exert their damaging effects?

In 2000, Mak et al. (150) employed gas chromatographic (GC)/MS analysis to monitor the levels of more than 20 different aldehydes in circulating arterial blood plasma samples collected from congestive heart failure (CHF) patients, and also those from age-matched participants with normal left ventricle (LV) function (*n* = 8 in each case). Aldehydic LOPs determined featured long- and short-chain *n*-alkanals, *trans*-2-alkenals, 4-hydroxy-*trans*-2-alkenals, and *trans,trans*-2,4-alkadienals, along with MDA. Mean blood plasma levels, or mean aldehyde concentration ranges for differential homologues within each aldehyde class, are available in Table 5 for these two groups of study participants, as indeed are ranges for the individual sampling values of these aldehyde classes determined in *n* = 36 French fry servings purchased from fast-food restaurants.

**Table 5:** Mean concentrations, or concentration ranges of mean values of aldehydes determined in the blood plasma of *n* = 8 patients with CHF and *n* = 8 age-matched normal LV function controls by a GC/MS technique [obtained from Agency for Toxic Substances and Disease Registry (150)].

	**Normal LV function Controls (nmol./L)**	**CHF Disease (nmol./L)**	**French Fries (μmol./kg)**
Long-Chain *n*-Alkanals (7)	69–573	42–339	19–560
Short-Chain *n*-Alkanals (1)	67	91	nd
*trans*-2-Alkenals (4)	106–527	163–874	0–430
4-Hydroxy-*trans*-2-Alkenals (4)	33–211	16–434	0.5–2.1 (38)
*trans,trans*-2,4-Alkadienals (2)	152–180	148–420	0–443
MDA	96	101	0–6^*^
Furfural	2,450	4,060	nd

*The bracketed numbers in the first (molecular classification) column refer to the number of aldehydes included for each classification specified for the blood plasma samples analysed. Also listed are the ranges of contents (μmol./kg) found for samples of French fry samples purchased from fast-food/take-out restaurants (long- and short-chain n-alkanals, trans-2-alkenals, and trans,trans-alka-2,4-dienals were determined by our ^1^H NMR analysis approach, but those for 4-hydroxy-trans-2-alkenals are as reported in Csallany et al. (65) using HPLC and HPLC/MS methods. However, both 4-hydroxy-trans-2-alkenals and furfural are also readily ^1^H-NMR-detectable and quantifiable*.

* *MDA was specifically determined by the method described in Grootveld et al. (45), which involved the reaction of thiobarbituric acid (TBA) with this dialdehyde to form a pink/red chromophoric derivative, but only subsequent to its relatively specific extraction into an aqueous medium (mean ± SD first extraction efficacy: 78 ± 2%). Adapted from Grootveld et al. (56) with permission*.

In Grootveld et al. (56), we reported a study involving a comparative statistical exploration of the above mean blood plasma aldehyde classification concentration patterns expressed as fractions of the total aldehyde level found in these samples, for both the above LV function control and CHF groups, to those of the same mean molar ratios of such classifications found in French fry portions purchased from fast-food restaurants (Table 5). The employment of such proportionate molecular ratios as variables for this study was considered to be appropriate in view of the expectation that they are less sensitive to the possible influences of demographic variables, e.g., study participant ages, genders, and body-mass indices (BMIs), etc., than the aldehyde class levels themselves.

Untransformed, raw blood plasma aldehyde measurements revealed that CHF patients had significantly higher levels of total aldehydic LOPs, along with those of a series of unsaturated ones, i.e., *trans*-2-alkenals and 4-hydroxy-*trans*-2-alkenals (the latter group featuring HHE and HNE), and furfural, than those of the normal LV function control group (150) [furfural is not a LOP, but a common food flavouring agent, which also displays potential genotoxic and carcinogenic properties, and is also hepatotoxic (151, 152)]. In contrast, the latter control group had significantly upregulated *n*-alkanal concentrations over those of CHF patients.

Following removal of the non-LOP furfural from the calculations, our reported investigation (56) found very similar proportionate contents of both *n*-alkanals and *trans*-2-alkenals in the potato chip and normal LV function blood plasma groups, and this observation suggested that such classes of aldehyde may be predominantly dietary-derived. If so, then the retention of their fractional concentration ratio *in vivo* may also be consistent with their differential, albeit lower molecular reactivities than those of 4-hydroxy-*trans*-2-alkenals and MDA following human ingestion. However, major differences were found between these two groups' fractional 4-hydroxy-*trans*-2-alkenal and MDA levels (with much higher proportionate concentrations in the blood plasma samples), and these indicated that such toxins may be generated from lipid peroxidation processes occurring *in vivo*, a postulate which is also supported by higher levels of the former class of aldehydes in the CHD group of patients.

Acetaldehyde is also detectable and monitorable in whole human blood and blood plasma. Indeed, in 1993, Helander et al. (153) investigated the distribution of acetaldehyde in its free and protein-bound forms. These experiments involved the precipitation of fresh whole blood with perchloric acid (HClO_4_); an aliquot of the crude sample was then employed for determining “total” acetaldehyde, whereas the clear supernatant arising therefrom was analysed for free “soluble” acetaldehyde. These researchers found that although this simple saturated aldehyde was usually below its lower limit of detection (0.2 μmol./L) in separated plasma, much greater levels were observed when whole blood served as an analytical matrix (>2.5 μmol./L)— ~70% of this was present as the HClO_4_-insoluble, protein-bound form. Notwithstanding, it appeared that some acetaldehyde was released from blood cells during the HClO_4_ precipitation stage of these experiments.

Acrolein, which also acts as a severe nasal and tracheobronchial airway irritant, is implicated in the development and pathobiology of chronic obstructive pulmonary disease (COPD) (100). Intriguingly, mean acrolein levels in blood plasma are in excess of 200 μg/L (*ca*. 3.5 μmol./L) in patients with heavy smoking histories in all stages (I-IV) of COPD disease, and almost. 300 μg/L (5.3 μmol./L) for those in its most severe stage IV classification; however, that for non-COPD disease smokers was 60 μg/L (~1 μmol./L) (154). Although much of this plasma acrolein presumably arises from the inhalation of tobacco cigarette smoke, a significant proportion may also be derived from the consumption of dietary sources of it (60). Additionally, from this study, the mean “free” acrolein levels in human lung tissue biopsy-derived supernatants were found to be *ca*. 4-fold greater for both COPD and smoking COPD patients over those of non-smoking controls. Moreover, immunohistochemistry staining of these tissues revealed that protein-bound acrolein was significantly expressed in both the COPD and non-COPD tobacco smoker groups, with little detectable therein in subjects who had never smoked. Corresponding studies have confirmed increased concentrations of this reactive aldehyde in the bronchoalveolar lavage fluid of COPD patients (155).

Could these residual aldehyde concentrations be considered toxicologically- or even clinically-significant? Notably, one recent report (156) found that low, conceivably non-toxic but physiologically-relevant doses of acrolein (0.5–1.0 μmol./L) significantly suppressed myogenic differentiation *in vitro*, a process which may occur through a mechanism involving Akt signalling inhibition. Furthermore, in mice, acrolein was found to induce muscle wasting, and also attenuated muscle regeneration. Therefore, such results provide evidence for acrolein serving as a significant risk factor for myogenesis and disease-related myopathy.

## Conclusions

PUFA-rich culinary oils in particular produce very high concentrations of hazardous LOPs when exposed to high-temperature frying practises: PUFAs are much more susceptible to thermally-induced peroxidation than MUFAs (7–9). In contrast, saturated fatty acids (SFAs) are very highly resistant to lipid peroxidation, and therefore should be recommended as one of the most select media for use in frying episodes. Likewise, oils containing high or very high contents of MUFAs should also be recommended (47), since lower or much lower amounts of LOPs are generated in such frying media than those found with PUFA-rich oils such as sunflower oil when exposed to high-temperature frying practises.

The potential contributions of toxic aldehydic LOPs to the pathogenesis and incidences of NCDs are supported by a plethora of evidence available, and a full outline of this is provided in Moumtaz et al. (47). One example is strong causal associations between the risk of coronary heart disease (CHD) and the recurrent consumption of fried food meals, specifically ≥4 times per week (157). Moreover, linkages between deep-fried food consumption and prostate cancer risk have been demonstrated (3), and a meta-analysis of published data found that an increased fried food intake engendered an estimated 35% enhanced risk of this condition (158).

As an additional input, the cardiovascular studies of Ismahil et al. (159) found that “Long-term oral exposure to acrolein, at an amount within the range of human unsaturated aldehyde intake, induces a phenotype of dilated cardiomyopathy in the mouse. Human exposure to acrolein may have analogous effects and raise considerations of an environmental, aldehyde-mediated basis for heart failure”.

Some “optimistic” members of the food industry and their associated researchers, i.e., the healthy PUFA-rich frying oil mindset, claim that aldehydes have a favourable contribution to the “fried food” aroma of French fries. However, strong linkages between the inhalation of cooking oil fumes (presumably including this aroma) and the development/incidence of lung cancer in non-smoking Chinese females, have been established (76–78). In Moumtaz et al. (47), it has already been stressed that the very high levels of aldehydes present in used, PUFA-rich frying oils, and which are directly transferable to fried foods, only represents the fraction remaining therein following their volatilisation during frying episodes; b.pts of a very high proportion of aldehydic LOPs are close to, lower, or much lower than that of standard frying temperature (180°C), as shown in Table 2. Astoundingly, total concentrations of α,β-unsaturated aldehydes remaining in such frying oils exposed to repeated frying episodes can sometimes exceed 50 mmol./kg (47). Therefore, in the absence of aldehyde-consuming chemical reactions in fried foods [which we suggest do occur in view of differences observed between the patterns and concentrations of aldehydic LOPs therein, and those found in corresponding frying oils (47)], human consumption of only a 1.00 g mass of such a peroxidised oil in this fried food form will yield an α,β-unsaturated aldehyde content of ≥50 μmoles, which again substantially exceeds the above WHO 9.36 μmole/day limit estimate.

If there was a substance or substances more toxic than paraquat in my food sources, and the amount there was potentially hazardous to human health, then I think I would want to know about it, thank you very much, rather than the issue being brushed aside as being too unimportant to consider. Currently, the EU has a maximum residue limit for paraquat in the majority of foodstuffs, which is sub-micromolar, i.e., 20 μg/kg (= 78 nmol./kg) (160). This limit is, of course, substantially lower than the concentrations of any of the above aldehyde classifications found in fried foods.

Although arguably present at lower levels, chemically-reactive dietary aldehydes in fried foods and used cooking oils are much more toxic, and have much broader toxicological profiles, than *trans*-fatty acids (*trans*-FAs) (56, 57); notably, intakes of the latter are currently largely dependent on whether or not the nations where they may be consumed have legislation in place to ban or restrict their adverse production, uses and human consumption rates/extents. However, secondary aldehydic LOPs are present in such food products at much higher concentrations than those of the food production contaminants acrylamide and *mono*-chloro-propanediols (MCPDs) (57), agents with highly documented toxicological and deleterious health properties.

The rigorous establishment of currently-unavailable BMDL_10_, ADI (TDI) and maximum human daily intake (MHDI) values for many dietary aldehydic LOPs is therefore a very important future requirement. To date, data available on these toxins is largely limited to agents arising as industrial contaminants and pollutants, notably acrolein, acetaldehyde and formaldehyde. Although there are some relevant data available on alternative aldehydes which are also dietary LOPs, for example deca-(*trans,trans*)-2,4-dienal (74), these are largely restricted to their commercial application as food flavouring agents, the added contents of which are much lower than those determined in thermally-stressed cooking oils and fried foods. These considerations are now of much clinical significance in view of major consumer concerns regarding the nutritional and health properties, both positive and negative, of contemporary foods and global dietary patterns.

Also urgently required is the performance of carefully designed nutritional and epidemiological trials to investigate relationships between the ingestion of dietary LOPs, especially those consumed in fried food sources, and the incidence, progression and severity of NCDs. Indeed, one notable feature of previous cohort trials focussed only on the intakes of differential types of acylglycerol fatty acids in diets surveyed, is that for the great majority of studies, no account whatsoever of whether or not sources of these lipids have been exposed to LOP-generating high-temperature frying or cooking episodes prior to their dietary ingestion. Indeed, most frequently the LOP contents of such consumed foods are completely neglected or ignored. Ideally, such proposed future trials should specifically be LOP-focussed.

## Author Contributions

The author confirms being the sole contributor of this work and has approved it for publication.

## Conflict of Interest

The author declares that the research was conducted in the absence of any commercial or financial relationships that could be construed as a potential conflict of interest.

## Publisher's Note

All claims expressed in this article are solely those of the authors and do not necessarily represent those of their affiliated organizations, or those of the publisher, the editors and the reviewers. Any product that may be evaluated in this article, or claim that may be made by its manufacturer, is not guaranteed or endorsed by the publisher.

## Footnotes

^1^ www.esds.ac.uk/findingData/snDescription.asp?sn$=$6533

^2^https://r.search.yahoo.com/_ylt=AwrIQhJlHYRg8McA2QR3Bwx.;%20ylu=Y29sbwMEcG9zAzEEdnRpZAMEc2VjA3Ny/RV=2/RE=1619299814/RO=10/RU=https%3a%2f%2f; https://www.statista.com/statistics/765075/consumption-percapita-from-oil-in-spain-by-kind/ (accessed April 30, 2021).

## References

[1] National Research Council (US) Committee on Technological Options to Improve the Nutritional Attributes of Animal Products. Designing Foods: Animal Product Options in the Marketplace. Washington, DC: National Academies Press (US) (1988). p. 4. Consumer Concerns and Animal Product Options. Available online at: https://www.ncbi.nlm.nih.gov/books/NBK218169/25032293

[2] Iqbal R Anand S Ounpuu S Islam S Zhang X Rangarajan S. Dietary patterns and the risk of acute myocardial infarction in 52 countries: results of the interheart study. Circulation. (2008) 118:1929–37. 10.1161/CIRCULATIONAHA.107.738716 18936332

[3] Stott-Miller M Neuhouser ML Stanford JL. Consumption of deep-fried foods and risk of prostate cancer. Prostate. (2013) 73:960–9. 10.1002/pros.22643 23335051 PMC3756514

[4] Choe E Min DB. Mechanisms and factors for edible oil oxidation. Comprehen Rev Food Sci Food Safety. (2006) 5:169–86. 10.1111/j.1541-4337.2006.00009.x31619649

[5] Haywood RM Claxson AWD Hawkes GE Richardson DP Naughton DP Coumbarides G. Detection of aldehydes and their conjugated hydroperoxydiene precursors in thermally-stressed culinary oils and fats: investigations using high resolution proton NMR spectroscopy. Free Rad Res. (1995) 22:441–82. 10.3109/10715769509147552 7633572

[6] Grootveld M Ruiz-Rodado V Silwood CJL. Detection, monitoring and deleterious health effects of lipid oxidation products generated in culinary oils during thermal stressing episodes. Inform Am Oil Chem Soc. (2014) 25:614–24. Available online at: http://hdl.handle.net/2086/11160

[7] Frankel EN. Volatile lipid oxidation-products. Prog Lipid Res. (1983) 22:1–33. 10.1016/0163-7827(83)90002-4 6306693

[8] Frankel EN. Lipid Oxidation. Dundee, Scotland: The Oily Press (1998). p. 9.

[9] Dobarganes MC Perez-Camino MC. Fatty acid composition: a useful tool for the determination of alteration level in heated fats. Rev Franc Corps Gras. (1988) 35:67–70.

[10] Claxson AWD Hawkes GE Richardson DP Naughton DP Haywood RM Chander CL. Generation of lipid peroxidation products in culinary oils and fats during episodes of thermal stressing: a high field ^1^H NMR study. FEBS Lett. (1994) 355:81–90. 10.1016/0014-5793(94)01147-8 7957968

[11] Guillén MD Uriarte PS. Contribution to further understanding of the evolution of sunflower oil submitted to frying temperature in a domestic fryer: study by ^1^H nuclear magnetic resonance. J Agric Food Chem. (2009) 57:7790–9. 10.1021/jf900510k 19663427

[12] Grootveld M Atherton MD Sheerin AN Hawkes J Blake DR Richens TE. In vivo absorption, metabolism, and urinary excretion of α,β-unsaturated aldehydes in experimental animals. Relevance to the development of cardiovascular diseases by the dietary ingestion of thermally-stressed polyunsaturate-rich culinary oils. J Clin Invest. (1998) 101:1210–8. 10.1172/JCI1314 9502761 PMC508674

[13] Penumetcha M Khan N Parthasarathy S. Dietary oxidized fatty acids: an atherogenic risk? J Lipid Res. (2000) 41:1473–80. 10.1016/S0022-2275(20)33460-X 10974055

[14] Staprãns I Rapp JH Pan XM Hardman DA Feingold KR. Oxidized lipids in the diet accelerate the development of fatty streaks in cholesterol-fed rabbits. Arterioscler Thromb Vasc Biol. (1996) 16:533–8. 10.1161/01.ATV.16.4.533 8624775

[15] Stavridis JC. Toxicity Carcinogenicity of Aldehydes. In: Stavridis JC, editors. Oxidation: The Cornerstone of Carcinogenesis. Oxidation Tobacco Smoke Carcinogenesis. A Relationship Between Cause Effect. London: Springer Science & Business Media UK Ltd (2007). p. 161–73. 10.1007/978-1-4020-6704-4_11

[16] Benigni R Passerini L Rodomonte A. Structure–activity relationships for the mutagenicity and carcinogenicity of simple and α-β unsaturated aldehydes. Environ Mol Mutagen. (2003) 42:136–43. 10.1002/em.10190 14556221

[17] Long EK Murphy TC Leiphora LJ Watt J Morrow JD Milne GL. Trans-4-hydroxy-2-hexenal is a neurotoxic product of docosahexaenoic (22:6; n-3) acid oxidation. J Neurochem. (2008) 105:714–24. 10.1111/j.1471-4159.2007.05175.x 18194211

[18] Grootveld M Silwood CJL Addis P Claxson A Bonet Serra B Viana M. Health effects of oxidised heated oils. Foodserv Res Int. (2001) 13:39–53. 10.1111/j.1745-4506.2001.tb00028.x26148922

[19] Benedetti A Ferrali M Casini AF Pieri S Comporti M. Foot-edema induced by carbonyl compounds originating from the peroxidation of liver microsomal lipids. Biochem Pharmacol. (1980) 29:121–4. 10.1016/0006-2952(80)90256-7 7362621

[20] Indart A Viana M Grootveld MC SiIwood CJL Sanchez-Vera I Bonet B. Teratogenic actions of thermally-stressed culinary oils in rats. Free Radic Res. (2002) 36:1051–8. 10.1080/1071576021000006716 12516875

[21] Jayaraj AP Rees KR Tovey FEI White JS. A molecular basis of peptic ulceration due to diet. Br J Exp Path. (1986) 67:149–55. 3753877 PMC2013067

[22] Yang Z Piironen V Lampi A-M. Epoxy and hydroxy fatty acids as non-volatile lipid oxidation products in oat. Food Chem. (2019) 295:82–93. 10.1016/j.foodchem.2019.05.052 31174813

[23] Zheng J Plopper C Lakritz J Storms DH Hammock BD. Leukotoxin-diol: a putative toxic mediator involved in acute respiratory distress syndrome. Am J Respir Cell Mol Biol. (2001) 25:434–8. 10.1165/ajrcmb.25.4.4104 11694448

[24] Brühl L Weisshaar R Matthäus B. Epoxy fatty acids in used frying fats and oils, edible oils and chocolate and their formation in oils during heating. Eur J Lipid Sci Technol. (2016) 118:425–34. 10.1002/ejlt.20150023525855820

[25] Xia W Budge SM. Techniques for the analysis of minor lipid oxidation products derived from triacylglycerols: epoxides, alcohols, and ketones. Compr Rev Food Sci Food Saf . (2017) 16:735–58. 10.1111/1541-4337.12276 33371569

[26] Velasco J Marmeset S Bordeaux G Rquez-Ruiz M Dobarganes C. Formation and evolution of monoepoxy fatty acids in thermoxidized olive and sunflower oils and quantitation in used frying oils from restaurants and fried-food outlets. J Agric Food Chem. (2004) 52:4438–43. 10.1021/jf030753f 15237949

[27] Mubiru E Jacxsens L Papastergiadis A Lachat C Shrestha K Rubel Mozumder NHM. Exposure assessment of epoxy fatty acids through consumption of specific foods available in Belgium. Food Addit Contam Part A Chem Anal Control Expo Risk Assess. (2017) 34:1000–11. 10.1080/19440049.2017.1310399 28349745

[28] Fankhauser-Noti A Fiselier K Biedermann-Brem S Grob K. Assessment of epoxidized soy bean oil (ESBO) migrating into foods: Comparison with ESBO-like epoxy fatty acids in our normal diet. Food Chem Toxicol. (2006) 44:1279–86. 10.1016/j.fct.2006.02.005 16600458

[29] Greene JF Newman JW Williamson KC Hammock BD. Toxicity of epoxy fatty acids and related compounds to cells expressing human soluble epoxide hydrolase. Chem Res Toxicol. (2000) 13:217–26. 10.1021/tx990162c 10775319

[30] Wilson R Fernie CE Scrimgeour CM Lyall K Smyth L Riemersma RA. Dietary epoxy fatty acids are absorbed in healthy women. Eur J Clin Invest. (2002) 32:79–83. 10.1046/j.1365-2362.2002.00951.x 11895453

[31] Schaich KM. Co-oxidations of oxidizing lipids: Reactions with proteins. In: Kamal-Eldin A Min D editors. Lipid Oxidation Pathways Vol. 2. AOCS Press (2008). p. 183–274.

[32] Nawar WW. Thermal degradation of lipids. A review. J Agric Food Chem. (1969) 17:18–21. 10.1021/jf60161a012

[33] Zhao Y Chen C Zhao B. Is oil temperature a key factor influencing air pollutant emissions from Chinese cooking? Atmos Environ. (2018) 193:190–7. 10.1016/j.atmosenv.2018.09.012

[34] Lawson H. Deep Fat frying. In: Lawson HW editor. Chapter 7. Food oils and Fats. New York, NY: Chapman and Hall (1995). p. 66–115. 10.1007/978-1-4757-2351-9_7

[35] Moreira RG Castell Perez ME Barrufet MA. Oil Absorption in fried food. In: Deep- Fat Frying; Fundamental and Applications. Gaithersburg, MD: Chapman & Hall Food Science Book (1999). p. 179–221.

[36] Yoon SH Jung MY Min DB. Effects of thermally oxidized triglycerides on the oxidative stability of soyabean oil. J Am Oil Chem Soc. (1988) 65:1652–6. 10.1007/BF02912571

[37] Tseng YC Moreira RG Sun X. Total frying use time effects on soybean oil deterioration and on tortilla chip quality. Intl J Food Sci Technol. (1996) 31:287–94. 10.1046/j.1365-2621.1996.00338.x

[38] Casimir MB David MB. Food Lipids Chemistry, Nutrition and Biotechnology, 4rth Edn, (2017). p. 205–218.

[39] Mert A Brajendra SK Price JPN Perez MJ Doll MK Evidence Evidence Contrary to the accepted diels- alder machinism in the thermal modification of vegetable oil. J Am Oil Chem Soc. (2012) 89:987–94. 10.1007/s11746-011-2002-x

[40] Lascaray L Mechanism Mechanism of fat splitting. Ind Eng Chem. (1949) 41:786–90. 10.1021/ie50472a025

[41] Chung J Lee J Choe E. Oxidative stability of soyabean and sesame oil mixture, beef, tallow and palm oil during frying of steamed noodles. Korean J Food Sci Technol. (2004) 30:288–92. 10.1111/j.1365-2621.2004.tb13652.x

[42] Romero A Cuesta C Sanchez Muniz FJ. Effects of oil replenishment during deep- fat turnover of fresh oil. J Am Oil Chem Soc. (1998) 70:1069–73.

[43] Blumenthal MM. A new look at the chemistry and physics of deep-fat frying. Food Technol. (1991) 45:68–71. 10.1016/0924-2244(91)90659-7

[44] Dimitra HP Vassiliki O Constantina T. The effects of process time tempersture on the accumulation of polar compounds in cottonseed oil during deep-fat frying, J Sci Food Agric. (2003) 83:314–9. 10.1002/jsfa.131425855820

[45] Grootveld M Percival BC Moumtaz S Gibson M Woodason K Akhtar A. Commentary: Iconoclastic reflections on the ‘safety' of polyunsaturated fatty acid-rich culinary frying oils: some cautions regarding the laboratory analysis and dietary ingestion of lipid oxidation product toxins. Appl Sci. (2021) 11:2351. 10.3390/app11052351

[46] Grootveld M Ruiz-Rodado V Silwood CJL. Detection, monitoring and deleterious health effects of lipid oxidation products generated in culinary oils during thermal stressing episodes. Inform AOCS. (2014) 25:614–24.

[47] Moumtaz S Percival BC Parmar D Grootveld KL Jansson P Grootveld. Toxic aldehyde generation in and food uptake from COs during frying practices: peroxidative resistance of a monounsaturate-rich algae oil. Sci Rep. (2019) 9:4125. 10.1038/s41598-019-39767-1 30858398 PMC6412032

[48] Wann AI Percival BC Woodason K Gibson M Vincent S Grootveld M. Comparative ^1^H NMR-based chemometric evaluations of the time-dependent generation of aldehydic lipid oxidation products in culinary oils exposed to laboratory-simulated shallow frying episodes: differential patterns observed for omega-3 fatty acid-containing soybean oils. Foods. (2021) 10:2481. 10.3390/foods10102481 34681530 PMC8535530

[49] Guillén MD Uriarte PS. Aldehydes contained in edible oils of a very diferent nature afer prolonged heating at frying temperature: presence of toxic oxygenated α,β unsaturated aldehydes. Food Chem. (2012) 131:915–26. 10.1016/j.foodchem.2011.09.079

[50] Martinez-Yusta A Goicoechea E Guillen MD. A review of thermo-oxidative degradation of food lipids studied by ^1^H NMR spectroscopy: influence of degradative conditions and food lipid nature. Compr Rev Food Sci Food Saf. (2014) 13:838–59. 10.1111/1541-4337.12090

[51] Almoselhy RIM Allam MH El-Kalyoubi MH El-Sharkawy AA. ^1^H NMR spectral analysis as a new aspect to evaluate the stability of some edible oils. Ann Agric Sci. (2014) 59:201–6. 10.1016/j.aoas.2014.11.006

[52] Feron VJ Til HP de Vrijer F Woutersen RA Cassee FR van Bladeren PJ. Aldehydes: occurrence, carcinogenic potential, mechanism of action and risk assessment. Mutat Res. (1991) 259:363–85. 10.1016/0165-1218(91)90128-9 2017217

[53] Gibson S Kurilich AC. The nutritional value of potatoes and potato products in the UK diet. Nutr Bull. (2013) 38:389–99. 10.1111/nbu.12057

[54] Markaverich BM Crowley JR Alejandro MA Shoulars K Casajuna N Mani S. Leukotoxin diols from ground corncob bedding disrupt estrus cyclicity in rats and stimulate MCF-7 breast cancer cell proliferation. Environ Health Perspect. (2005) 113:1698–704. 10.1289/ehp.8231 16330350 PMC1314908

[55] Public Health England (2016). Collection. National Diet and Nutrition Survey. The National Diet and Nutrition Survey Assesses the Diet, Nutrient Intake and Nutritional Status of the General Population of the UK.

[56] Grootveld M Percival BC Leenders J Wilson P. Potential adverse public health effects afforded by the ingestion of dietary lipid oxidation product toxins: Significance of fried food sources. Nutrients. (2020) 12:974. 10.3390/nu12040974 32244669 PMC7254282

[57] Grootveld M Percival BC Grootveld KL. Chronic non-communicable disease risks presented by lipid oxidation products in fried foods. Hepatobiliary Surg Nutr. (2018) 7:305–12. 10.21037/hbsn.2018.04.01 30221162 PMC6131264

[58] Guallar-Castillón P Rodríguez-Artalejo F Lopez-Garcia E León-Muñoz LM Pilar Amiano P Ardanaz E. Consumption of fried foods and risk of coronary heart disease: Spanish cohort of the European Prospective Investigation into cancer and nutrition study. BMJ. (2012) 344:e363. 10.1136/bmj.e363 22275385 PMC3265571

[59] European Union Risk Assessment Report. Acrolein CAS No. 107-02-8, EINECS-No. 203-453-4, Institute for Health and Consumer Protection, European Chemicals Bureau (existing substances), 1^st^ Priority List, Volume 7, EUR 19728EN (2001). Environment and quality of life series. ISBN-92-894-0501-5.

[60] Wang GW Guo Y Vondriska TM Zhang J Zhang S Tsai LL. Acrolein consumption exacerbates myocardial ischemic injury and blocks nitric oxide-induced PKCepsilon signaling and cardioprotection. J Mol Cell Cardiol. (2008) 44:1016–22. 10.1016/j.yjmcc.2008.03.020 18468618

[61] Percival BC Wann A Zbasnik R Schlegel V Edgar M Zhang J. Evaluations of the peroxidative susceptibilities of cod liver oils by a ^1^H NMR analysis strategy: peroxidative resistivity of a natural collagenous and biogenic amine-rich fermented product. Nutrients. (2020) 12:753. 10.3390/nu12030753 32178350 PMC7146420

[62] Boskou G Salta FN Chiou A Troullidou E. Andrikopoulos NK. Content of trans,trans-2,4-decadienal in deep-fried and pan-fried potatoes. Eur J Lipid Sci Technol. (2006) 108:109–15. 10.1002/ejlt.20050023625855820

[63] Le Gresley A Ampem G De Mars S Grootveld M Naughton DP. “Real-world” evaluation of lipid oxidation products and trace metals in French fries from two chain fast-food restaurants. Front Nutr. (2021) 8:620952. 10.3389/fnut.2021.620952 33614697 PMC7892784

[64] Esterbauer H Ertl A Scholz N. The reaction of cysteine with α,β-unsaturated aldehydes. Tetrahedron. (1976) 32:285–9. 10.1016/0040-4020(76)87015-9

[65] Csallany AS Han I Shoeman DW Chen C Yuan J. 4-Hydroxynonenal (HNE), a toxic aldehyde in french fries from fast food restaurants. J Am Oil Chem Soc. (2015) 92:1413–9. 10.1007/s11746-015-2699-z

[66] Wang L Csallany S Kerr BJ Shurson GC Chen C. Kinetics of forming aldehydes in frying oils and their distribution in French fries revealed by LC–MS-based chemometrics. Agric Food Chem. (2016) 64:3881–9. 10.1021/acs.jafc.6b01127 27128101

[67] LoPachin RM Gavin T. Molecular mechanisms of aldehyde toxicity: a chemical perspective. Chem Res Toxicol. (2014) 27:1081–91. 10.1021/tx5001046 24911545 PMC4106693

[68] Ahmed Laskar A Younus H. Aldehyde toxicity and metabolism: the role of aldehyde dehydrogenases in detoxification, drug resistance and carcinogenesis. Drug Metab Rev. (2019) 51:42–64. 10.1080/03602532.2018.1555587 30514131

[69] Acceptable Daily Intakes (ADI) for Agricultural and Veterinary Chemicals Used in Food Producing Crops or Animals. Edition 4/2017 current as of 31 December 2017. Commonwealth of Australia 2017. ISSN 1446-1412.

[70] Health Health Implications of Acrylamide in Food Report of a Joint FAO/WHO Consultation WHO Headquarters Geneva Switzerland 25-27 June, 2002. Issued by the World Health Organization in collaboration with the Food and Agriculture Organization of the United Nations Food Safety Programme Department of Protection of the Human Environment. World Health Organisation. Available online at: https://apps.who.int/iris/bitstream/handle/10665/42563/9241562188.pdf;sequence=1

[71] World Health Organization (WHO). Human Health Risk Assessment Toolkit: Chemical. (2010). Available online at: http://www.who.int/ipcs/methods/harmonization/areas/ra_toolkit/en/ (accessed February 18, 2020).

[72] ATSDR. Agency for Toxic Substances Disease Registry, Toxicological Profile for Acrolein. U.S. Department of Health and Human Services (2007). Available online at: https://www.atsdr.cdc.gov/toxprofiles/tp.asp?id=557&tid=102> (accessed February 18, 2020).

[73] Lachenmeier DW Kanteres F Rehm J. Carcinogenicity of acetaldehyde in alcoholic beverages: risk assessment outside ethanol metabolism. Addiction. (2009) 104:533–50. 10.1111/j.1360-0443.2009.02516.x 19335652

[74] Williams G. WHO Food Additives Series: 52. Aliphatic, alicyclic, linear alpha,beta-unstaurated di- and trienals and related alcohols, acids and esters. Prepared by the Sixty-first meeting of the Joint FAO/WHO Expert Committee on Food Additives (JECFA). World Health Organization, Geneva, 2004. International program on chemical safety (IPCS) INCHEM Home. Available online at: http://www.inchem.org/documents/jecfa/jecmono/v52je14.htm

[75] WHO Library Cataloguing-in-Publication Data Safety evaluation of certain food additives/prepared by the sixty-third meeting of the Joint FAO/WHO Expert Committee on Food Additives (JEFCA). (WHO food additives series; 54) 1. Food additives — toxicity 2. Flavoring agents — toxicity 3. Food contamination 4. Risk assessment I. Joint FAO/WHO Expert Committee on Food Additives. Meeting (63rd: 2004, Geneva, Switzerland II. Series. ISBN 92 4 166054 6 (NLM classification: WA 712) ISSN 0300-0923

[76] Zhong L Goldberg MS Gao YT Jin F. Lung cancer and indoor air pollution arising from Chinese-style cooking among non-smoking women living in Shanghai, China. Epidemiology. (1999) 10:488–94. 10.1097/00001648-199909000-00005 10468420

[77] Xue Y Jiang Y Jin S Li Y. Association between cooking oil fume exposure and lung cancer among Chinese nonsmoking women: a meta-analysis. Onco Targets Ther. (2016) 9:2987–92. 10.2147/OTT.S104334 27284248 PMC4881732

[78] Hecht SS Koh WP Wang R Chen M Carmella SG Murphy SE. Elevated levels of mercapturic acids of acrolein and crotonaldehyde in the urine of Chinese women in Singapore who regularly cook at home. PLoS ONE. (2015) 10:e0120023. 10.1371/journal.pone.0120023 25807518 PMC4373935

[79] Silano V Bolognesi C Castle L Cravedi J-P Engel K-H Fowler P. Scientific Opinion on Flavouring Group Evaluation 226 Revision 1 (FGE.226 Rev1): consideration of genotoxicity data on -unsaturated aldehyde from chemical subgroup 1.1.1(b) of FGE.19 EFSA Panel on Food Contact Materials, Enzymes, Flavourings and Processing Aids (CEF). ADOPTED (2017). 10.2903/j.efsa.2017.4847 PMC7010128 32625501

[80] Grant RL Jenkins AF. Use of *in vivo* and *in vitro* data to derive a chronic reference value for crotonaldehyde based on relative potency to acrolein. J Toxicol Environ Health B Crit Rev. (2015) 18:327–43. 10.1080/10937404.2015.1081574 26580244 PMC4706029

[81] Wolfe G Rodwin M French J Parker G. Thirteen week subchronic toxicity study of crotonaldehyde (CA) in F344 rats and B6C3F1 mice. Toxicologist. (1987) 7:209.

[82] Wiseman J. Optimizing the role of fats in diet formulation. In: Proceedings of the Australian Poultry Science Symposium. (1999). p. 8–15.

[83] Lykkesfeldt J Svendsen O. Oxidants and antioxidants in disease: oxidative stress in farm animals. Vet J. (2007) 173:502–11. 10.1016/j.tvjl.2006.06.005 16914330

[84] Grootveld M Percival BC Gibson M Osman Y Edgar M Molinari M. Progress in low-field benchtop NMR spectroscopy in chemical and biochemical analysis. Anal Chim Acta. (2019) 1067:11–30. 10.1016/j.aca.2019.02.026 31047142

[85] Shurson GC Kerr BJ Hanson AR. Evaluating the quality of feed fats and oils and their effects on pig growth performance. J Animal Sci Biotechnol. (2015) 6:10. 10.1186/s40104-015-0005-4 25844168 PMC4384276

[86] Belitz HD Grosch W Schieberle P: Lipids. In: Belitz HD Grosch W editors. Food Chemistry. (Schieberle P. Berlin: Springer).

[87] Gray R Robinson H. Free fatty acids and rancidity in relation to animal by-product protein concentrates. Poult Sci. (1941) 20:36–41. 10.3382/ps.0200036

[88] Azain MJ. Fat in swine nutrition. In: Lewis AJ Southern LL, editors. Swine Nutrition. Boca Raton: CRC Press (2001) p. 95–106. 10.1201/9781420041842.ch6

[89] DeRouchey J Hancock J Hines R Maloney C Lee D Cao H. Effects of rancidity and free fatty acids in choice white grease on growth performance and nutrient digestibility in weanling pigs. J Anim Sci. (2004) 82:2937–44. 10.2527/2004.82102937x 15484945

[90] Liu P Chen C Kerr BJ Weber TE Johnston LJ Shurson GC. Influence of thermally-oxidized vegetable oils and animal fats on growth performance, liver gene expression, and liver and serum cholesterol and triglycerides in young pigs. J Anim Sci. (2014) 92:2960–70. 10.2527/jas.2012-5709 24879755

[91] Yuan I Kerr BJ Curry SM Chen CC. Identification of C9-C11 unsaturated aldehydes as prediction markers of growth and feed intake for non-ruminant animals fed oxidized soybean oil. J Anim Sci Biotechnol. (2020) 11:49. 10.1186/s40104-020-00451-4 32411370 PMC7206673

[92] Selim S Hussein E. Production performance, egg quality, blood biochemical constituents, egg yolk lipid profile and lipid peroxidation of laying hens fed sugar beet pulp. Food Chem. (2020) 310:125864. 10.1016/j.foodchem.2019.125864 31780225

[93] Woutersen RA Appel MJ van Garderen-Hoetmer A Wijnands MV. Dietary fat and carcinogenesis. Mutat Res. (1999) 443:111–27. 10.1016/S1383-5742(99)00014-9 10415435

[94] Dung CH Wu SC Yen GC. Genotoxicity and oxidative stress of the mutagenic compounds formed in fumes of heated soybean oil, sunflower oil and lard. Toxicol In Vitro. (2006) 20:439–47. 10.1016/j.tiv.2005.08.019 16216463

[95] Garrido-Polonio C García-Linares MC García-Arias MT López-Varela S García-Fernández MC Terpstra AH. Thermally oxidized sunflower-seed oil increases liver and serum peroxidation and modifies lipoprotein composition in rats. Br J Nutr. (2004) 92:257–65. 10.1079/BJN20041174 15333157

[96] Hageman G Verhagen H Chutte B. Biological effects of short-term feeding to rats of repeatedly used deep-frying fats in relation to fat mutagen content. J Food Chem Toxicol. (1991) 29:689–98. 10.1016/0278-6915(91)90127-S 1959823

[97] Vandemoortele A Heynderickx PM Leloup L De Meulenaer B. Kinetic modeling of malondialdehyde reactivity in oil to simulate actual malondialdehyde formation upon lipid oxidation. Food Res Int. (2021) 140:110063. 10.1016/j.foodres.2020.110063 33648286

[98] Songur A Sarsilmaz M Ozen O Sahin S Koken R Zararsiz I. The effects of inhaled formaldehyde on oxidant and antioxidant systems of rat cerebellum during the postnatal development process. Toxicol Mech Methods. (2008) 18:569–74. 10.1080/15376510701555288 20020855

[99] Matveychuk D Dursun SM Wood PL Baker GB. Reactive aldehydes and neurodegenerative disorders. Bull Clin Psychopharm. (2011) 21:277–88. 10.5455/bcp.19691231040000

[100] Fitzmaurice AG Rhodes SL Lulla A Murphy NP Lam HA O'Donnell KC. Aldehyde dehydrogenase inhibition as a pathogenic mechanism in Parkinson Disease. Proc Natl Acad Sci USA. (2013) 110:636–41. 10.1073/pnas.1220399110 23267077 PMC3545765

[101] Vaglini F Pardini C Viaggi C Bartoli C Dinucci D Corsini GU. Involvement of cytochrome P450 2E1 in the 1-methyl-4-phenyl-1,2,3,6- tetrahydropyridine-induced mouse model of Parkinson's disease. J Neurochem. (2004) 91:285–98. 10.1111/j.1471-4159.2004.02720.x 15447662

[102] Jurnak F. The pivotal role of aldehyde toxicity in autism spectrum disorder: the therapeutic potential of micronutrient supplementation. Nutr Metab Insights. (2015) 8:57–77. 10.4137/NMI.S29531 27330305 PMC4910734

[103] Leikauf GD. United States Environmental Protection Agency (EPA) final report: Inhalation of aldehydes and effects of breathing. EPA Grant Number R828112CO49.

[104] Venkata RP Subramanyam R. Evaluation of the deleterious health effects of consumption of repeatedly heated vegetable oil. Toxicol Rep. (2016) 3:636–43. 10.1016/j.toxrep.2016.08.003 28959587 PMC5616019

[105] Osim EE Owu DU Etta KM. Arterial pressure and lipid profile in rats following chronic ingestion of palm oil diets. Afr J Med Sci. (1996) 25:335–40. 9532303

[106] Leong XF Aishah A Nor Aini U Das S Jaarin K. Heated palm oil causes rise in blood pressure and cardiac changes in heart muscle in experimental rats. Arch Med Med Res. (2008) 39:567–72. 10.1016/j.arcmed.2008.04.009 18662587

[107] Owu DU Orie NN Osim EE. Altered responses of isolated aortic smooth muscle following chronic ingestion of palm oil diets in rats. Afr J Med Med Sci. (1997) 26:83–6. 10895239

[108] Falade AO Oboh G Oluwaseun A Oluwatoyin A Odubanjo V. Consumption of thermally oxidized palm oil diets alters biochemical indices in rats, Beni-Suef Univ J Basic Appl Sci. (2015) 4:150–6. 10.1016/j.bjbas.2015.05.009

[109] World Health Organization, Geneva, Gomes R Meek ME. Concise International Chemical Assessment Document 43 ACROLEIN (2002).

[110] Acceptable Acceptable daily intakes for agricultural and veterinary chemicals: current as of 31 March 2016 Australian Australian Government Department of Health (Commonwealth of Australia 2016). The Office of Chemical Safety, Department of Health, MDP 71, GPO Box 9848, CANBERRA ACT2601 (2016).

[111] Aggett P Nordberg GF Nordberg M. Chapter 14–Essential Metals: Assessing Risks from Deficiency and Toxicity. In: Nordberg GF Fowler BA Nordberg M, editors. Handbook on the Toxicology of Metals (Fourth Edition). Cambridge, MA: Academic Press (2015). p. 281–97. 10.1016/B978-0-444-59453-2.00014-7

[112] Parent RA Caravello HE Hoberman AH. Reproductive study of acrolein on two generations of rats. Fundam Appl Toxicol. (1992) 19:228–37. 10.1093/toxsci/19.2.228 1516780

[113] Vandenberg LN Welshons WV vom Saal FS Toutain P-L Myers JP. Should oral gavage be abandoned in toxicity testing of endocrine disruptors? Environ Health. (2014) 13:46. 10.1186/1476-069X-13-46 24961440 PMC4069342

[114] Paasche A Schiller M Schirmeister T Engels B. Mechanistic study of the reaction of thiol-containing enzymes with alpha,beta-unsaturated carbonyl substrates by computation and chemoassays. Chem Med Chem. (2010) 5:869–80. 10.1002/cmdc.201000020 20401893 PMC7162195

[115] Hébert CD Yuan J Dieter MP. Comparison of the toxicity of cinnamaldehyde when administered by microencapsulation in feed or by corn oil gavage. Food Chem Toxicol. (1994) 32:1107–15. 10.1016/0278-6915(94)90126-0 7813982

[116] EFSA (European Food Safety Authority) and WHO (World Health Organization) (2016). Review of the Threshold of Toxicological Concern (TTC) approach and development of new TTC decision tree. EFSA supporting publication. (2016): EN-1006. 50 pp.

[117] Roberts DW Aptula A Schultz TW Shen J Api AM Bhatia S. A practical guidance for Cramer class determination. Regul Toxicol Pharmacol. (2015) 73:971–84. 10.1016/j.yrtph.2015.09.017 26382611

[118] Eckl PM Bresgen N. Review article: genotoxicity of lipid oxidation compounds. Free Radic Biol Med. (2017) 111:244–52. 10.1016/j.freeradbiomed.2017.02.002 28167130

[119] Cho Y Song M Kim TS Ryu JC. DNA Methylome analysis of saturated aliphatic aldehydes in pulmonary toxicity. Sci Rep. (2018) 8:10497. 10.1038/s41598-018-28813-z 30002397 PMC6043580

[120] Nadasi E Varjas T Pajor L Ember I. Carcinogenic potential of trans-2-hexenal is based on epigenetic effect. In Vivo. (2005) 19:559–62. 15875776

[121] Kiwamoto R Spenkelink A Rietjens I.MCM Punt A. A physiologically based in silico model for trans-2-hexenal detoxification and DNA adduct formation in human including interindividual variation indicates efficient detoxification and a negligible genotoxicity risk. Arch Toxicol. (2013) 87:1725–37. 10.1007/s00204-013-1091-8 23864024

[122] U.S. Environmental Protection Agency. Integrated Risk Information System (IRIS) on Acrolein. National Center for Environmental Assessment, Office of Research and Development, Washington, DC (2003). Available online at: https://www.epa.gov/sites/production/files/2016-08/documents/acrolein.pdf

[123] Chung F-L Tanaka T Hecht SS. Induction of liver tumors in F344 rats by crotonaldehyde. Cancer Res. (1986) 46:1285–9. 3002613

[124] Eder E Budiawan. Cancer risk assessment for the environmental mutagen and carcinogen crotonaldehyde on the basis of TD_50_ and comparison with 1,N^2^-propanodeoxyguanosine adduct levels. Cancer Epidemiol Biomarkers Prev. (2001) 10:883–8. 11489755

[125] Eder E Schuler D Budiawan. Cancer risk assessment for crotonaldehyde and 2-hexenal: an approach. IARC Sci Publ. (1999) 150:219–32. PMID: 10626223. 10626223

[126] Lutz WK. Quantitative evaluation of DNA binding data for risk estimation and for classification of direct binding carcinogens. J Cancer Res Clin Oncol. (1986) 112:85–91. 10.1007/BF00404387 3095332 PMC12252959

[127] Salaspuro M. Commentary: acetaldehyde a cumulative carcinogen in humans. Addiction. (2009) 104:551–3. 10.1111/j.1360-0443.2009.02546.x 19335653

[128] Salaspuro M. Acetaldehyde and gastric cancer. J Digest Dis. (2011) 12:51–9. 10.1111/j.1751-2980.2011.00480.x 21401890

[129] Seitz HK Stickel F. Acetaldehyde as an underestimated risk factor for cancer development: role of genetics in ethanol metabolism. Genes Nutr. (2009) 5:121–8. 10.1007/s12263-009-0154-1 19847467 PMC2885165

[130] Dellarco VLA. Mutagenicity assessment of acetaldehyde. Mutat Res. (1988) 195:1–20. 10.1016/0165-1110(88)90013-9 3122032

[131] Theruvathu JA Jaruga P Nath RG Dizdaroglu M Brooks PJ. Polyamines stimulate the formation of mutagenic 1,N^2^-propanodeoxyguanosine adducts from acetaldehyde. Nucleic Acid Res. (2005) 33:3513–20. 10.1093/nar/gki661 15972793 PMC1156964

[132] Moghe A Ghare S Lamoreau B Moghe A Ghare S Lamoreau B. Molecular mechanisms of acrolein toxicity: relevance to human disease. Toxicol Sci. (2015) 143:242–255. 10.1093/toxsci/kfu233 25628402 PMC4306719

[133] Stevens JF Maier CS. Acrolein: sources, metabolism, and biomolecular interactions relevant to human health and disease. (2008) Mol Nutr Food Res. 52:7–25. 10.1002/mnfr.200700412 18203133 PMC2423340

[134] Eder E Scheckenbach S Deininger C Hoffman C. The possible role of alpha, beta-unsaturated carbonyl compounds in mutagenesis and carcinogenesis. Toxicol Lett. (1993) 67:87–103. 10.1016/0378-4274(93)90048-3 8451772

[135] International Agency for Research on Cancer. IARC Monographs on the Evaluation of Carcinogenic Risks to Humans Volume 88 (2006): Formaldehyde, 2-Butoxyethanol and 1-tert-Butoxypropan-2-ol. (2004). Available online at: http://monographs.iarc.fr/ENG/Monographs/vol88/index.phpExit_Disclaimer PMC4781641 17366697

[136] National Toxicology Program. Report on Carcinogens, Twelfth Edition. Department of Health and Human Services, Public Health Service, National Toxicology Program (2011). Available online at: http://ntp.niehs.nih.gov/go/roc12

[137] National Toxicology Program. Toxicology and Carcinogenesis Studies of Malonaldehyde, Sodium Salt. Technical Report. (1988) 331:5–13. 12732905

[138] Basu AK Marne ÂLJ. Molecular requirements for the mutagenicity of malondialdehyde and related acroleins. Cancer Res. (1984) 44:2848–2854. 6372997

[139] Singer B Muriseli H. The Role of Cyclic Nucleic Acid Adducts in Carcinogenesis and Mutagenesis. Vol. 70. Lyon, France: IARC Scientific Publications. International Agency for Research on Cancer (1986).3793179

[140] Smith RA Cohen SM Lawson TA. Acrolein mutagenicity in the V79 assay. Carcinogenesis. (1990) 11:497–8. 10.1093/carcin/11.3.497 2311195

[141] Marne ÂLJ Hurd HK. Hollstein MC Levin DE. Naturally occurring carbonyl compounds are mutagens in *Salmonella* tester strain TA 104. Mutat Res. (1985) 748:25–34. 10.1016/0027-5107(85)90204-0 3881660

[142] Cohen SM Garland EM St. John M Okamura T Smith RA. Acrolein initiates rat urinary bladder carcinogenesis. Cancer Res. (1992) 52:3577–3581. 1617627

[143] Tan SLW Chadha S Liu Y Gabasova E Perera D Ahmed K. A Class of environmental and endogenous toxins induces *BRCA2* haploinsufficiency and genome instability. Cell. (2017) 169:1105–18. 10.1016/j.cell.2017.05.010 28575672 PMC5457488

[144] Pontel LB Rosado IV Burgos-Barragan G Pontel LB Rosado IV Burgos-Barragan. Endogenous formaldehyde is a hematopoietic stem cell genotoxin and metabolic carcinogen. Molec Cell. (2015) 60:177–88. 10.1016/j.molcel.2015.08.020 26412304 PMC4595711

[145] Srivastava M Singh J George K Bhui Y. Shukla Genotoxic and carcinogenic risks associated with the consumption of repeatedly boiled sunflower oil. J Agric Food Chem. (2010) 58:11179–86. 10.1021/jf102651n 20886885

[146] Weng M-w Lee H-W Park S-Y Hu Y Wang HT Chen LC. Aldehydes are the predominant forces inducing DNA damage and inhibiting DNA repair in tobacco smoke carcinogenesis. Proc Natl Acad Sci USA. (2018) 115:E6152–61; 10.1073/pnas.1804869115 29915082 PMC6142211

[147] van Andel I Sleijffers A Schenk E. Adverse Health Effects of Cigarette Smoke: Aldehydes Crotonaldehyde, Butyraldehyde, Hexanal, And Malonaldehyde. RIVM Report 340630002/ (2006).

[148] Marques MM Beland FA Lachenmeier DW Phillips DH Chung F-L Dorman DC. Carcinogenicity of acrolein, crotonaldehyde, and arecoline. Lancet Oncol. (2021) 22:9–20. 10.1016/S1470-2045(20)30727-0 33248467

[149] Agency for Toxic Substances Disease Registry. Toxicological Profile for Acrolein. (2007). Available online at: http://www.atsdr.cdc.gov/ToxProfiles/tp124.pdf (accessed April 31, 2018).

[150] Mak S Lehotay DC Yazdanpanah M Azevedo ER Liu PP Newton GE. Unsaturated aldehydes including 4-OH-nonenal are elevated in patients with congestive heart failure. J Card Fail. (2000) 6:109–114. 10.1016/S1071-9164(00)90012-5 10908084

[151] Furfural (CAS 98-01-1). Carcinogenic Potency Project (2021).

[152] Irwin R. NTP Technical Report on the Toxicology and carcinogenesis studies of furfural (CAS NO. 98-01-1) in F344/N RATS B6C3F1 mice (gavage studies) (Report). U.S. Department of Health and Human Services (1990). 12692654

[153] Helander A Lowenmo C Johansson M. Distribution of acetaldehyde in human blood: effects of ethanol and treatment with disulfiram. Alcohol Alcoholism. (1993) 28:461–8. 8397528

[154] Yasuo M Droma Y Kitaguchi Y Ito M Imamura H Kawakubo M. The relationship between acrolein and oxidative stress in COPD: in systemic plasma and in local lung tissue. Int J Chron Obstruct Pulmon Dis. (2019) 14:1527–37. 10.2147/COPD.S208633 31371938 PMC6636184

[155] Bein K Leikauf G. Acrolein-A pulmonary hazard. Molec Nutr Food Res. (2011) 55:1342–60. 10.1002/mnfr.201100279 21994168

[156] Chen HJ Wang CC Chan DC Chiu CY Yang RS Liu SH. Adverse effects of acrolein, a ubiquitous environmental toxicant, on muscle regeneration and mass. J Cachexia, Sarcopenia Musc. (2019) 10:165–176. 10.1002/jcsm.12362 30378754 PMC6438343

[157] Gadiraju TV Patel Y Gaziano JM Djoussé L. Fried food consumption and cardiovascular health: A review of current evidence. Nutrients. (2015) 7:8424–30. 10.3390/nu7105404 26457715 PMC4632424

[158] Lippi G Mattiuzzi C. Fried food and prostate cancer risk: systematic review and meta-analysis. Internat J Food Sci Nutr. (2015) 66:587–9. 10.3109/09637486.2015.1056111 26114920

[159] Ismahil MA Hamid T Haberzettl P Ismahil MA Hamid T Haberzettl P. Chronic oral exposure to the aldehyde pollutant acrolein induces dilated cardiomyopathy. Am J Physiol-Heart and Circulat Physiol. (2011) 301:H2050–60 10.1152/ajpheart.00120.2011 21908791 PMC3213964

[160] IPCS International Programme On Chemical Safety Health and Safety Guide No. 51. Paraquat Health And Safety Guide United Nations Environment Programme International Labour Organisation World Health Organization World Health Organization. Geneva (1991). Available online at: inchem.org/documents/hsg/hsg/hsg051.htm

